# A Mitosome With Distinct Metabolism in the Uncultured Protist Parasite *Paramikrocytos canceri* (Rhizaria, Ascetosporea)

**DOI:** 10.1093/gbe/evad022

**Published:** 2023-02-15

**Authors:** Ioana Onuț-Brännström, Courtney W Stairs, Karla Iveth Aguilera Campos, Markus Hiltunen Thorén, Thijs J G Ettema, Patrick J Keeling, David Bass, Fabien Burki

**Affiliations:** Department of Organismal Biology, Program in Systematic Biology, Uppsala University, Uppsala, Sweden; Microbiology Research Group, Department of Biology, Lund University, Lund, Sweden; Microbiology Research Group, Department of Biology, Lund University, Lund, Sweden; Department of Organismal Biology, Program in Systematic Biology, Uppsala University, Uppsala, Sweden; Laboratory of Microbiology, Wageningen University and Research, Wageningen, The Netherlands; Department of Botany, University of British Columbia, Vancouver, British Columbia, Canada; International Centre of Excellence for Aquatic Animal Health, Centre for Environment, Fisheries and Aquaculture Science (Cefas), Weymouth, United Kingdom; Department of Life Sciences, The Natural History Museum, London, United Kingdom; Sustainable Aquaculture Futures, Biosciences, College of Life and Environmental Sciences, University of Exeter, Exeter, United Kingdom; Department of Organismal Biology, Program in Systematic Biology, Uppsala University, Uppsala, Sweden; Science for Life Laboratory, Uppsala University, Uppsala, Sweden

**Keywords:** mitochondrial evolution, mitosome, mikrocytids, *Paramikrocytos canceri*, Ascetosporea, rhizaria, protists, parasites

## Abstract

Ascetosporea are endoparasites of marine invertebrates that include economically important pathogens of aquaculture species. Owing to their often-minuscule cell sizes, strict intracellular lifestyle, lack of cultured representatives and minimal availability of molecular data, these unicellular parasites remain poorly studied. Here, we sequenced and assembled the genome and transcriptome of *Paramikrocytos canceri*, an endoparasite isolated from the European edible crab *Cancer pagurus*. Using bioinformatic predictions, we show that *P. canceri* likely possesses a mitochondrion-related organelle (MRO) with highly reduced metabolism, resembling the mitosomes of other parasites but with key differences. Like other mitosomes, this MRO is predicted to have reduced metabolic capacity and lack an organellar genome and function in iron–sulfur cluster (ISC) pathway-mediated Fe–S cluster biosynthesis. However, the MRO in *P. canceri* is uniquely predicted to produce ATP via a partial glycolytic pathway and synthesize phospholipids de novo through the CDP-DAG pathway. Heterologous gene expression confirmed that proteins from the ISC and CDP-DAG pathways retain mitochondrial targeting sequences that are recognized by yeast mitochondria. This represents a unique combination of metabolic pathways in an MRO, including the first reported case of a mitosome-like organelle able to synthesize phospholipids de novo. Some of these phospholipids, such as phosphatidylserine, are vital in other protist endoparasites that invade their host through apoptotic mimicry.

**Significance**

Mitochondria-related organelles (MROs) carry out subsets of functions otherwise found in aerobic mitochondria. These organelles have evolved in response to life in low-oxygen environments and/or as an adaptation to a parasitic lifestyle. In order to obtain a better understanding of the full functional spectrum of MROs, discovering and characterizing the metabolic pathways present in MROs from unexplored parts of the eukaryotic tree of life is critical. Here, we use new genomic data from the uncultured parasite *Paramikrocytos canceri*, which causes mortality in edible crabs, to characterize an extremely reduced MRO in the supergroup Rhizaria. Genome mining and heterologous gene expression in yeast revealed an organelle resembling a mitosome but with unique differences currently not known in other mitosomes in that it might produce ATP via glycolysis and synthesize phospholipids de novo. Overall, this study reveals unique features in a mitosome and broadens the functional diversity of MROs from understudied parasites.

## Introduction

Mitochondria are best known as the powerhouse organelles supplying eukaryotic cells with energy in the form of ATP via oxidative phosphorylation where oxygen serves as the terminal electron acceptor (Van der Giezen and Tovar 2005; Müller et al. 2012; Makiuchi and Nozaki 2014; Stairs et al. 2015; Roger et al. 2017; Gawryluk and Stairs 2021). Mitochondria harbor several other fundamental cellular metabolisms ranging from Fe–S cluster biogenesis to the synthesis of phospholipids, quinones, steroids, amino acid, and nucleotides (Van der Giezen and Tovar 2005; Santos et al. 2018). From these complex aerobic mitochondria, a diverse range of related organelles with reduced functions (mitochondrion-related organelles; MROs) have evolved across the tree of eukaryotic life in anaerobic and microaerophilic lineages of unicellular organisms (protists) (Müller et al. 2012; Makiuchi and Nozaki 2014; Stairs et al. 2015; Roger et al. 2017; Santos et al. 2018; Gawryluk and Stairs 2021). The functional diversity of MROs covers a continuum with no clear boundaries (Müller et al. 2012; Makiuchi and Nozaki 2014; Stairs et al. 2015; Roger et al. 2017; Santos et al. 2018; Gawryluk and Stairs 2021), but a few main “classes” have been proposed (Müller et al. 2012). Along the most reduced end of the spectrum are the genome-less hydrogenosomes that produce hydrogen, have lost the respiratory chain and oxidative phosphorylation, and generate ATP exclusively via substrate-level phosphorylation (Müller et al. 2012; Stairs et al. 2015; Leger et al. 2016; Stairs et al. 2021). Even more reduced are the mitosomes, which do not produce ATP, but continue to be involved in the biosynthesis of Fe–S clusters (Tovar et al. 2003; Goldberg et al. 2008; Mi-ichi et al. 2009; Jedelský et al. 2011) or sulfate activation (Mi-ichi et al. 2009). The near ubiquity of Fe–S cluster biosynthesis pathways even in the most reduced MROs has led to the suggestion that this function is one of the fundamental roles of the MROs, although other currently poorly characterized functions such as the production of formate and methylated folate species were recently observed (Zítek et al. 2022).

MROs have been best studied in parasitic lineages of medical importance, including the hydrogenosomes of trichomonads (e.g., *Trichomonas vaginalis* (Müer 1993), or the mitosomes of microsporidia (e.g., *Encephalitozoon cuniculi* (Katinka et al. 2001), diplomonads (e.g., *Giardia intestinalis* (Tovar et al. 2003)) and amoebozoa (e.g., *Entamoeba histolytica* (Mai et al. 1999)). However, the known functional diversity of MROs has greatly expanded in recent years from genomic investigations of hitherto enigmatic and often neglected parasitic (Burki et al. 2013; Santos et al. 2018; Mathur et al. 2021; Salomaki et al. 2021), commensal, as well as free-living protists (Stairs et al. 2014; Stairs et al. 2021, Zítek et al. 2022). MROs have been reported in nearly all major eukaryotic supergroups, demonstrating remarkable convergent adaptations to life in low-oxygen or host environments. Yet, the characterization of new MROs, especially from poorly sampled supergroups, remains an important task that will enable us to better understand the tremendous plasticity of mitochondrial functions and the parallel reductive evolution of these organelles.

Ascetosporea is a group of poorly known unicellular parasites that belongs to Rhizaria, which is also of the least investigated supergroups of eukaryotes (Burki and Keeling 2014; Bass et al. 2019; Biard 2023). Ascetosporean parasites infect a vast range of aquatic invertebrates, including many commercially important shellfish species such as mussels and oysters, often causing economic losses and limiting trade due to biosecurity legislation (Mourton et al. 1992; Hartikainen et al. 2013; Hartikainen et al. 2014). It is a relatively diverse group, with about 50 described species and several hundreds more lineages known only from environmental sequences. These parasites are challenging to study, because they are generally very small (<5 µm), intracellular, and not easily separable from host tissues. More importantly, no cultures are available, thereby limiting the scope of experiments that can be performed. Despite these difficulties, a handful of transcriptomes of Ascetosporea have been produced, including that of the Pacific oyster parasite *Mikrocytos mackini* by sequencing cDNA of semipurified isolates prepared from laboratory-infected hosts (Burki et al. 2013). The phylogenomic analysis of *M. mackini* revealed that this parasite is extremely fast-evolving, displaying the longest branch in a phylogenetic tree among all included eukaryotes. Moreover, *M. mackini* is predicted to possess a highly reduced MRO only functioning in protein folding and Fe–S cluster generation via the ISC pathway. This was the first report of an MRO in Rhizaria (Burki et al. 2013). Following this discovery, a less reduced hydrogen-producing mitochondrion was described in another rhizarian lineage, the microaerophilic and free-living cercomonad *Brevimastigomonas motovehiculus* (Gawryluk et al. 2016).

In this study, we hypothesized that other ascetosporean parasites have adapted to intracellular lifestyle by reducing mitochondrial functions. To expand our knowledge of the functional range of mitochondria in Ascetosporea, we analyzed the genetic complement of *Paramikrocytos canceri,* a close relative of *M. mackini* (Hartikainen et al. 2014). This parasite was discovered as the agent of an emerging disease in juvenile crabs in the UK, causing hypertrophy of the antennal gland and bladder of reproductively immature crabs (Bateman et al. 2011; Thrupp et al. 2013; Hartikainen et al. 2014). In addition to crabs, targeted molecular surveys showed that *P. canceri* is in fact present in a range of shoreline invertebrates, although its impact on these diverse hosts remains uncertain (Hartikainen et al. 2014). Microscopic examinations of infected crab tissue showed numerous uni- and multi-nucleate plasmodial cells invading the host cytoplasm. Transmission electron microscopy images revealed that the uninucleate stages are often found near the host's mitochondria, while the plasmodial stages contain an abundance of minuscule (approx. 0.5 nm in diameter), spherical double membrane-bounded organelle which were described as putative MROs of unknown functions (Hartikainen et al. 2014).

Here, we present genomic and transcriptomic data for *P. canceri* isolated from host-infected tissues collected directly from the environment. Reads corresponding to *P. canceri* were extracted from a heterogeneous mixture of host and microbiome using a comprehensive bioinformatic workflow and were assembled into the first ascetosporean genome with high completeness. We used this genome to reconstruct the energy metabolism of *P. canceri* and compare that to its close relative *M. mackini*. Altogether, we confirm the previous TEM observations of the presence of an MRO in *P. canceri* (Hartikainen et al. 2014), inferring similar functions as in the mitosome of *M. mackini*, which probably originated in their common ancestor. However, we find that this newly described organelle is predicted to perform at least two metabolic pathways that have not been described in other reduced mitosomes: de novo phospholipid synthesis and parts of glycolysis.

## Results

### Genome Sequencing, Assembly, and Quality of *Paramikrocytos canceri* Genomes and Transcriptome

**Fig. 1.**
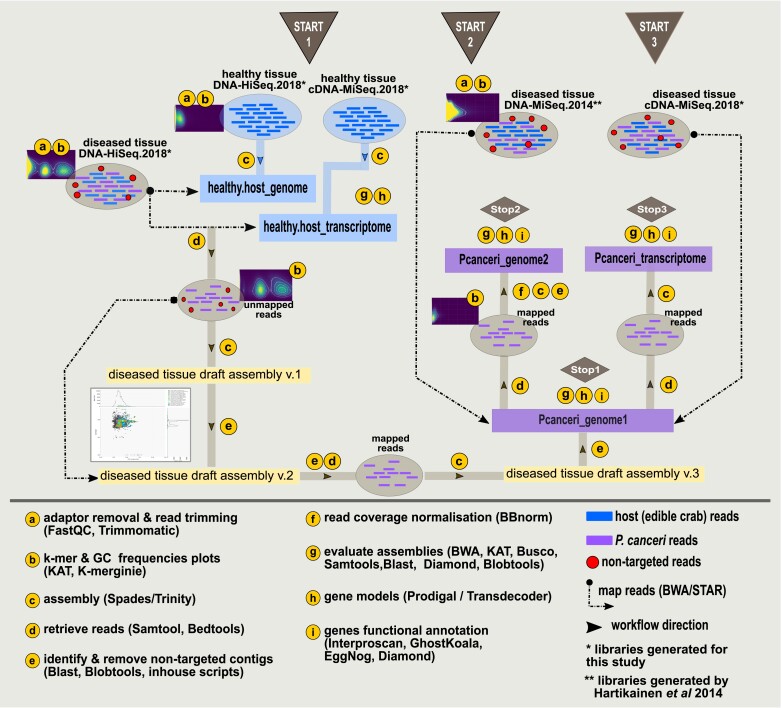
**Bioinformatic workflow to assemble the genomic and transcriptomic data of *P. canceri.*** The letters in yellow circles indicate the methodology used for each step of the workflow, while the arrows show the direction of the workflow from ‘start’ to ‘stop’. The Illumina libraries and the decontamination pipeline to obtain each of the assemblies used in this study are shown. START 1 depicts the workflow that generated the first *P. canceri* genome assembly (Pcanceri_genome1) from the diseased DNA HiSeq library that were generated for this study. In START 2, *P. canceri* reads were retrieved from the diseased DNA HiSeq library generated by Hartikainen et al 2014 (Hartikainen et al. 2014) and assembled in the second *P. canceri* genome assembly (Pcanceri_genome2). The transcriptome of *P. canceri* was assembled following the workflow in START 3 (Pcanceri_transcriptome).

To investigate the energy and mitochondrial metabolism of *P. canceri*, we sequenced DNA and cDNA libraries generated from both heavily infected antennal glands of diseased juvenile crabs and claw muscle tissues isolated from a healthy individual (supplementary fig. S1, Supplementary Material online). The “healthy tissue”“ DNA library was used to subtract the host reads from mixed reads by mapping the DNA library of the “diseased tissue”“ to the assembly generated from the DNA reads of the healthy crab (fig. 1—healthy.host_genome box). Based on the BlobTools workflow that uses GC content, read coverage, and taxonomic identity of the contigs obtained by blast searches (Laetsch and Blaxter 2017), we discarded the non-target reads (e.g., prokaryotes, fungi, crab) from the diseased tissue DNA library (fig. 1—START 1 of the workflow). The resulting dataset highly enriched in *P. canceri* reads was assembled to yield a first genome assembly of the parasite (*Pcanceri*_genome1) (fig. 1—START 1 of the workflow). This assembly was used as reference to retrieve *P. canceri* reads from a previously published metagenomic Illumina library of a diseased crab tissue (Hartikainen et al. 2014), and generate a second genome assembly (*Pcanceri_*genome2) (fig. 1—START 2 of the workflow). Finally, the reads of the diseased tissue RNA library that mapped to the *P. canceri* reference genome were assembled in the *P. canceri* transcriptome (fig. 1—START 3 of the workflow). All statistics for the intermediate and final assemblies are summarized in Table 1. The two *P. canceri* genome assemblies showed similar N50 (∼6800 bp), GC content (∼30%) and assembly size (∼13 Mb) (Table 1). According to BUSCO values, genome completeness was estimated to be 16.1% and 23.1% using the nucleotide and protein sequences from the gene models, respectively (Table 1; supplementary fig. S2, panel A Supplementary material online). Considering the lack of BUSCO protein sets for organisms related to *P. canceri*, together with the highly divergent nature of the genome (that reduces the detection sensitivity of similarity searches), these values more probably reflect the lack of publicly available data than very partial genomes. Comparably low values (19.4% and 30% using the nucleotide and protein sequences, respectively—Table 1) were also observed for the transcriptome of *M. mackini* (Table 1), or for published genomes of other protists (e.g., *Monocercomonoides exilis*: BUSCO score = 34%) (Karnkowska et al. 2016).

**Table 1:** Assembly Statistics

Assembly	Name of the assembly on ENA/NCBI and in phylogenies	Accession number	GC content	N50	Assembly size	number of contigs/transcripts	BUSCO score genome/trancriptome	fraction of reads that map onto the assembled genome	Read coverage	gene models	BUSCO scores predicted proteoms	number of predicted peptides	Functional annotation
healthy.host_genome	Meta.Cpag.2018.HiSeq_Ge	ERZ3058081	40.16	2108	4.5 GB	274,666	61%	97.32%	24.95	not predicted	-	-	not predicted
healthy.host_transcriptome	Meta.Cpag.2018.MiSeq_Tr	ERZ3062799	44.28	708	18.95 MB	48,899	58.1%	99.93%	60.00	TransDecoder v.5.3.0	59.1%	15,894	not predicted
diseased.host_metagenome	Meta.diseased.2018.HiSeq_Ge	ERZ3062800	39.56	2105	3.98 GB	272,597	58.8%	98.24%	43.11	Prodigal v.2.6.3	-	597,526	not predicted
diseased.host_transcriptome	Meta.diseased.2018.MiSeq_Tr	ERZ3065136	44.38	1008	17.02 MB	30,486	66.33%	99.58%	135.33	TransDecoder v.5.3.0	71%	13,686	not predicted
Pcanceri_genome 1	Pcan.2018.HiSeq_Ge	ERZ16272710	30.06	6806	12.69 MB	3113	16.1%	100%	717.13	Prodigal v.2.6.3	23.1%	8201	predicted
Pcanceri_transcriptome 1	Pcan.2018.MiSeq_Tr	HBWA01	39.59	1456	3.44 MB	4437	18.8%	99.86%	426.89	TransDecoder v.5.3.0	23.8%	2768	predicted
Pcanceri_genome 2	Pcan.2014.Miseq_Ge	ERZ16272508	30.45	6231	13.09 MB	4069	16.8%	99.93%	39.87	Prodigal v.2.6.3	21.4%	13,103	not predicted
Mmackini_transcriptome	Mmackini.GAHX01000001.1_Tr	GAHX01000001.1	36.20	1047	4.74 MB	7234	19.4%	see Burki et al 2013	448.845	TransDecoder v.5.3.0	30%	7234	predicted

The assembly were deposited on ENA (European Nucleotide Archive Databases) and their accession numbers can be consulted in column 3. The average GC content, the size of the genome assemblies, and the N50 was calculated with Quast. The transcriptome N50, the size of the transcriptome assemblies and number of transcripts were calculated with Trinity. The average read coverage for each assembly was calculated with Samtools as the number of mapped bases divided by the assembly size. The eukaryotic database euk_9 was used to calculate the BUSCO values. The functional annotation was predicted with BLAST suit v. 2.9.0+, InterProScan v.5.30-69.0, and eggNOG-mapper v2.

In addition to BUSCO estimates, we used three alternative methods to assess the *P. canceri* genome completeness. First, we looked at the fraction of reads that mapped onto the assembled genomes and the average read coverage for each assembly (Table 1). The reference assembly of *P. canceri* (*P. canceri*_genome1) has a high read coverage (above 700X; Table 1), and the entire read library was integrated in the assembly (100% of reads mapped onto the assembled genome—Table 1). We further compared the k-mer frequency spectra of the *P. canceri* reference genome and its respective read library (supplementary fig. S2, panel B Supplementary material online). Most of the read k-mers were assembled once and only a very low fraction of rare read k-mers is missing from the *P. canceri* reference assembly (see black and red frequency spectra from supplementary fig. S2, panel B Supplementary material online). The high read coverage and high proportion of read k-mers singularly assembled suggest that *P. canceri* genome is well recovered and not over assembled. As a second evaluation of genome completeness, we mapped *P. canceri* transcripts (only those that could be confidently attributed to *P. canceri*, i.e., not derived from host or other identifiable contaminants) to the *P. canceri* reference genome assembly. A total of 81.3% clean transcripts mapped back to the *P. canceri* reference genome. The remaining unmapped transcripts all have undetectable homology against the *nr* database (using an threshold of e-value < 1e-10) and consist mostly of short tandem simple repeats. The high proportion of mapped transcripts indicates a high recovery of genes for this assembly. As a last evaluation of assembly completeness, we used a set of 127 highly conserved marker genes previously identified in *M. mackini* (Burki et al. 2013) to search for homologues in the *P. canceri* genomes, and recovered all 127 genes. Using most of these genes for a concatenated phylogenomic analysis, we confirmed the sister relationship of *P. canceri* and *M. mackini* (supplementary fig. S3, Supplementary Material online). Taken together, these analyses indicate high completeness of both *P. canceri* assemblies despite the low BUSCO values and fairly high assembly fragmentation as a result of short-read sequencing.

### *Paramikrocytos canceri* is Predicted to Have a Mitosome-like Organelle that Functions in Fe–S Cluster Synthesis, Glycolysis, and *de novo* Phospholipid Synthesis

**Fig. 2.**
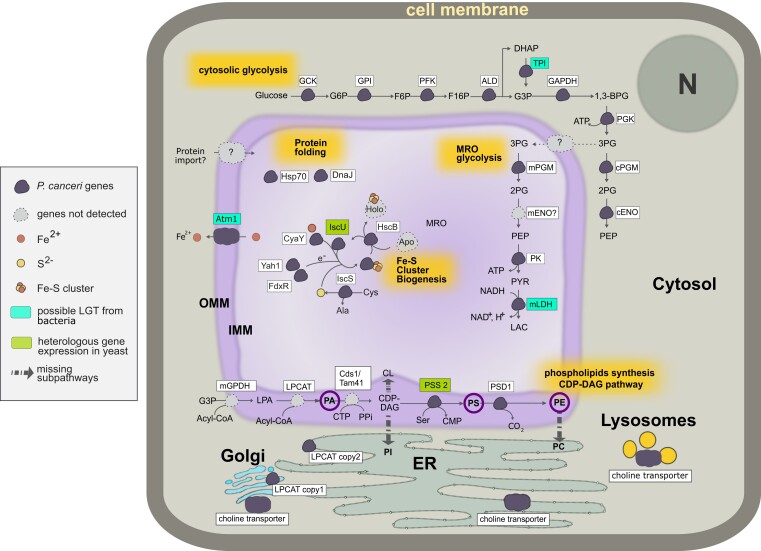
**Schematic representation of a *P. canceri* mitosome and the mitochondrial targeted pathways.** Metabolic pathways: Proteins are represented as small clouds and their name is highlighted in the white boxes. Purple clouds represent genes that were detected in *P. canceri* genomes and/or transcriptomes, while grey clouds are those that are presumed to be present but not identified. ‘?’ indicate proteins where mitochondrial provenance could not be confirmed with phylogenetic methods or MTS prediction. Abbreviations: Glycolysis: glucokinase (GCK), glucose-6-phosphate (G6P), phosphoglucose isomerase (GPI), fructose-6-phosphate (F6P), phosphofructokinase (PFK), fructose-1,6-biphosphate (F16P), fructose biphosphate aldolase (ALD), dihydroxyacetone phosphate (DHAP), triosephosphate isomerase (TPI), glyceraldehyde 3-phosphate (G3P), glyceraldehyde 3-phosphate dehydrogenase (GAPDH), 1,3-bisphosphoglycerate (1,3-BPG), phosphoglycerate kinase (PGK), 3-phosphoglycerate (3PG), cytosolic phosphoglycerate mutase (cPGM), 2-phosphoglycerate (2PG), cytosolic enolase (cENO), phosphoenolpyruvate (PEP), mitosomal phosphoglycerate mutase (mPGM), mitosomal enolase (mENO), pyruvate kinase (PK), pyruvate (PYR), mitosomal D-lactate dehydrogenase (mLDH), lactate (LAC); protein folding machinery: Hsp70, Hsp90 and DnaJ; Fe-S cluster biogenesis: ISC-associated transport protein (Atm1), frataxin (CyaY), yeast adrenodoxin homolog (Yah1), cysteine (Cys) desulfurase (IscS), alanine (Ala), Fe-S cluster chaperone (HscB), Iron-Sulfur Cluster Assembly Enzyme (IscU); CDP-DAG pathway: mitosomal glycerol-3-phosphate dehydrogenase (mGPDH), lysophosphatidic acid (LPA), LPA acyltransferase (LPCAT), phosphatidic acid (PA) CDP-diacylglycerol synthase 1 (Cds1), phosphatidic acid cytidylyltransferase (Tam41), CTP (cytidine triphosphate), PPi (Pyrophosphate), cytidine diphosphate diacylglycerol (CDP-DAG), serine (Ser), cytidine monophosphate (CMP), phosphatidylserine synthase 2 (PSS2), phosphatidylserine (PS), phosphatidylserine decarboxylase (PSD1).

We used our genome and transcriptome assemblies to predict the mitochondrial proteome of *P. canceri*. Using sequence homology, hidden Markov models, phylogenetic analysis and targeting signal predictions, we found at least 14 proteins likely to function in the mitochondrial compartment (fig. 2). We identified components of the ISC pathway for Fe–S cluster biosynthesis, chaperone proteins, and the ATP-binding cassette transporter ATM1 that carries the byproduct of the pathway (Fe^2+^) into to the cytoplasm (fig. 2, and supplementary Table S1, Supplementary Material online). Like for mitosomes in other organisms, we did not detect genes encoding proteins for the electron transport chain, ATP synthase, mitochondrial genome maintenance, and the Krebs cycle. As previously reported in the analysis of *M. mackini*, we also did not identify any ATP transporters or TIM/TOM translocon proteins (Burki et al. 2013). This suggests that TIM/TOM proteins are absent in both species or too divergent to be detected with current homology-based approaches.

In addition to the ISC pathway common to most mitosomes, we found that the MRO in *P. canceri* likely harbors two other pathways not known in such reduced organelles. One is glycolysis, for which we identified genes encoding a complete cytosolic glycolysis pathway but, unexpectedly, found two enzymes predicted to contain an MTS (fig. 2, supplementary Table S1, Supplementary Material online). The first enzyme is the pay-off phase cofactor-independent phosphoglycerate mutase (PGM), which is present in two copies in *P. canceri* with one copy containing an MTS. The sister species *M. mackini* also encodes two copies of *pgm* gene but neither contain a MTS. The second glycolytic enzyme predicted to be targeted to the MRO in *P. canceri* is pyruvate kinase (PK). Additionally, in both *P. canceri* genomes, we identified a D-lactate dehydrogenase (*ldh*) gene uniquely containing a 51-amino acid N-terminal extension predicted to encode an MTS (fig. 2, supplementary fig. S4A and Table S1, Supplementary Material online). Interestingly, phylogenetic analysis indicates a clear bacterial origin (Clostridia) of this gene (supplementary fig. S5, Supplementary Material online –mLDH phylogeny), which is contained in a scaffold (11,963 bp) in genome 1 that is contiguous (supported by mate pair information) and in synteny with a shorter scaffold (5377 bp) in genome 2 (supplementary fig. S4B, Supplementary Material online). The closest predicted neighboring gene to *ldh* on both scaffolds has no homology in GenBank, but a clear eukaryotic ribosomal protein (rpl30) is encoded on the scaffold in *P. canceri*_genome 1. Taken together, these observations suggest that in *P. canceri*, the last steps of the pay-off phase of glycolysis (from PGM to PK) and LDH (Nývltová et al. 2015; Glancy et al. 2020) may localize to the MRO and could generate ATP, reoxidized NAD+, and lactate, and that the mitochondrial *ldh* may have been acquired by lateral transfer possibly from Clostridia.

The third metabolic pathway likely functioning in the MRO of *P. canceri* is responsible for the de novo synthesis of phospholipids via the mitochondrial CDP-DAG pathway. In *P. canceri* (and *M. mackini*), we identified genes encoding three out of the five enzymes involved in the synthesis of phosphatidic acid, phosphatidylserine (PS), and phosphatidylethanolamine (PE), each with a predicted MTS (fig. 2 and supplementary Table S1, Supplementary Material online); these three enzymes are lysophosphatidic acid-acyltransferase (LPCAT), phosphatidylserine decarboxylase 1 (PSD1), and phosphatidylserine synthase 2 (PSS 2). In contrast, we did not detect any genes encoding proteins necessary for the synthesis of the rest of the phospholipid through the CDP-DAG pathway, that is phosphatidylcholine (PC), cardiolipin (CL), and phosphatidylinositol (PI) (fig. 2). The other main pathway for phospholipid synthesis in eukaryotes, the Kennedy pathway in the endoplasmic reticulum (ER) that uses exogenous phospholipid, was partial. Only one enzyme (choline/ethanolamine phosphotransferase) was detected in *P. canceri* and *M. mackini*, suggesting that these parasites are not able to generate PC nor PE from exogenous choline or ethanolamine. Yet, we were able to identify four choline transporters (predicted to function in the Golgi apparatus, ER, and lysosome) which suggest that the parasite could import choline from the host and distribute choline throughout its cell (fig. 2, supplementary Table S1, Supplementary Material online).

### Heterologous Expression of Fluorescently Tagged *P. canceri* Supports the *in Silico* Predictions

**Fig. 3.**
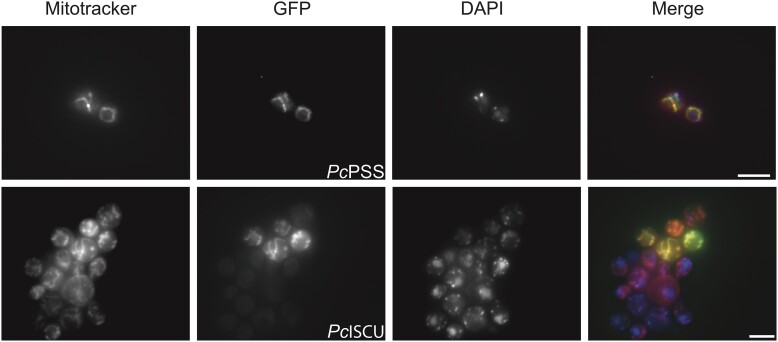
**Heterologous expressed green fluorescent protein (GFP) fusion proteins of *P. canceri* PSS and ISCU localize to yeast mitochondria.** Yeast cells expressing PSS-GFP (top, green) and ISCU-GFP (bottom, green) were stained with mitochondrion-reactive stain Mitotracker CMXRos (red) and DNA stain DAPI (blue) and visualized by fluorescent microscopy. Not all yeast cells are transformed. Images represent a composite of multiple Z-slices (see methods for details). Scale bars represent 5 µm.

To further assess the localization of some of the putative MRO proteins, GFP fusion protein constructs of PSS (from the CDP-DAG pathway), mPGM (from the glycolytic pathway), and IscU (from the Fe–S biosynthesis pathway) were expressed in yeast. GFP-labeled PSS and IscU co-localized with the mitoreactive stain Mitotracker strongly suggesting the proteins contained targeting signals that are recognized by the mitochondrial protein import machinery of yeast (fig. 3, supplementary fig. S6, Supplementary Material online). Attempts to express the mPGM (from glycolytic pathway) resulted in yeast with abnormal cell biology. Nevertheless, heterologous expression of fluorescently tagged *P. canceri* proteins involved in Fe–S cluster and phospholipid biosynthesis localized to yeast mitochondria brings further evidence supporting our bioinformatic predictions.

## Discussion

We report the first genome and transcriptome data for the parasite *P. canceri* from infected crabs collected directly from the host environment. Although poorly studied, these small, intracellular, and uncultivatable parasites are part of a large undescribed diversity of ascetosporean parasites of aquatic invertebrates that likely plays important roles in controlling natural populations (Hartikainen et al. 2014; Garcia et al. 2018). These data represent one of a very few genomic datasets for Ascetosporea as a whole and, to our knowledge, the most complete genome assembly for the group. Using phylogenomic analysis of 125 marker proteins, we demonstrate the sister relationship between *M. mackini* and *P. canceri* (supplementary fig. S3, Supplementary Material online). We further investigated the mitochondrial metabolism in *P. canceri* and found a very reduced set of functions indicative of an MRO supporting the available microscopic observations of small double membrane-bound organelles in this parasite (Hartikainen et al. 2014). This highly reduced MRO is predicted to function in Fe–S cluster synthesis via the ISC pathway, a characteristic shared amongst almost all mitosomes known to date. However, unlike other mitosomes, this organelle is predicted to (i) harbor part of the energy pay-off phase of glycolysis and (ii) synthesize phospholipids de novo through the CDP-DAG pathway (fig. 2, supplementary Table S1, Supplementary Material online). We also show that at least one component of the CDP-DAG pathway retains a putative mitochondrial localization signal that can be recognized by yeast cells in a heterologous expression system (fig. 3, supplementary fig. S6, Supplementary Material online). Collectively, these predicted features suggest that the MRO in *P. canceri* closely resembles a mitosome but with a distinct metabolism. This is the second example of a mitosome-like organelle in Rhizaria, following the description of a mitosome in the closely related parasite *M. mackini* (Burki et al. 2013).

The MRO in *P. canceri* is the first example of a mitosome-like organelle predicted to enclose a partial glycolysis. To our knowledge, such organelle-localized glycolysis has only been reported in functionally more “complex“ MROs or aerobic mitochondria from other eukaryotes. In some stramenopiles, for example, all genes involved in the pay-off phase of glycolysis exist in two copies, each with a predicted cytosolic and mitochondrial subcellular localization (Liaud et al. 2000; Abrahamian et al. 2017; Río Bártulos et al. 2018). Furthermore, the mitochondrial localized triphosphate isomerase (TPI) and glyceraldehyde 3-phosphate dehydrogenase (GAPDH) of stramenopiles are fused into a single protein equipped with an MTS. Similar fusion proteins containing clear N-terminal extensions have also been identified in other eukaryotes such as the cercozoan *Paulinella chromatophora* and the apuzozoan *Thecamonas trahens* (Nakayama et al. 2012). A partially duplicated glycolytic pathway was also predicted in the cercozoan *B. motovehiculus* (Gawryluk et al. 2016) and the ciliate *Tetrahymena thermophila* (Smith et al. 2007). The localization of the terminal steps of glycolysis in a mitosome could represent a way to obtain the necessary ATP for organelle-localized functions (e.g., protein folding). Other mitosome-containing organisms have solved the issue of ATP import in various ways. In the diplomonad *G. intestinalis*, ATP is produced in the parasite's cytosol through an extended glycolytic pathway and the arginine dihydrolase pathway, and imported into the organelle via an unknown transporter (Tovar et al. 2003; Jedelský et al. 2011). In microsporidia, ATP is directly harvested from the host cytoplasm and transferred through nucleoside transporter proteins into the mitosome (Tsaousis et al. 2008). In *Entamoeba histolytica*, glycolysis-derived ATP is imported from the cytoplasm via an ATP/ADP transporter from the mitochondrial carrier family (Chan et al. 2005). As for *M. mackini*, we could not detect genes encoding putative ATP transporters in *P. canceri* and neither PGM nor PK contain a mitochondrial targeting signal. However, we note that both of the *M. mackini* gene models predicted to encode PGM and PK are incomplete (supplementary Table S1, Supplementary Material online). Nevertheless they form well supported clades with the *P. canceri* proteins predicted to function in the MRO (supplementary fig. S5, Supplementary Material online—PGM and PK phylogenies). Moreover, like *P. canceri*, *M. mackini* encodes two copies of PGM. This suggests that *M. mackini* may also have a part of glycolysis localized in the MRO.

Based on the above results, we hypothesize that the relocation of the ATP-yielding pyruvate kinase reaction to the mitosome would allow ATP to be synthesized in the organelle rather than relying solely on ATP import from cytoplasmic pathways. However, because we did not detect a mitochondrial enolase or obvious nucleotide transporters in *P. canceri* that would be necessary for importing ADP/AMP for the pyruvate kinase reaction, it remains to be explained how the substrates for pyruvate kinase–phosphoenol pyruvate and ADP–are imported into the organelle. The mitosomes might also scavenge host-derived ATP, especially given microscopy evidence that show both *P. canceri* and *M. mackini* often found near host mitochondria (Burki et al. 2013; Hartikainen et al. 2014). Although not confirmed by the bioinformatic predictions, one of the identified ABC transporters (supplementary Table S1, Supplementary Material online) could be responsible for the host-derived ATP import into the parasite cells.

Another uncommon characteristic of the mitosome in *P. canceri* (and *M. mackini*) is the predicted capacity to synthesize phospholipids (fig. 2). Phospholipids are essential components of cellular membranes (Carman and Han 2011; Kay and Fairn 2019; Acoba et al. 2020) and are vital for many metabolic processes such as apoptosis (Amara and Mercer 2015), the electron transport chain Calzada et al. 2019), and cellular signaling (Carman and Han 2011). In some intracellular parasites, phospholipids are also important for immune evasion, host cell invasion, and nutrient acquisition (Vial et al. 2003; Campbell et al. 2013; Ramakrishnan et al. 2013; Wanderley et al. 2020). The biosynthesis of phospholipids and role of mitochondria therein have been best studied in model organisms such as yeast (Carman and Han 2011), but little is known of the contribution of MROs in parasites to these pathways. Experiments with radiolabeled phospholipids showed that phosphatidylethanolamine (PE) and PC localize to the mitosomes in the microsporidian *E. cuniculi* (El Alaoui et al. 2001) and the diplomonad *G. intestinalis* (Yichoy et al. 2010). Furthermore, the *pss* and *psd* genes from the CDP-DAG pathway were amplified by RT–PCR and sequenced in *E. cuniculi* (El Alaoui et al. 2001) and identified in the genomes of two other microsporidia (Campbell et al. 2013), although neither study specifies the subcellular location for their protein products. Collectively, these observations suggest that even though the role of MROs in phospholipid synthesis is understudied, MROs might play a more general role in phospholipid de novo synthesis, expanding the functional repertoire of reduced organelles such as mitosomes.

The possible involvement of mitosomes in de novo phospholipid synthesis in parasites is interesting because these compounds are critical for membrane structural integrity and cell signaling (Vance and Steenbergen 2005; Calianese and Birge 2020). Phosphatidylserine (PS), for example, signals the immune system to initialize phagocytosis and the anti-inflammatory response when expressed on the outer surface of apoptotic cells (El-Hani 2012; Amara and Mercer 2015; Wanderley et al. 2020). Some protistan endoparasites employ apoptotic mimicry to invade the host (Wanderley et al. 2020). By expressing high amounts of PS on its cell surface, such parasites will phenotypically look like a host apoptotic cell and will therefore be targeted for ingestion by phagocytes. Once phagocytosed, the parasite can propagate within the host cell without triggering the immune system. Invertebrates, including crabs, rely on their innate immune system which is constituted by cellular and humoral immune responses (Johnson 1987). Previous investigations showed that during *P. canceri* infection, host tubule cells that are infiltrated by *P. canceri* plasmodial cells become apoptotic. Yet, no immune response seems to be triggered in the host. The heavily infected crab haematocytes (known to be involved in the crab immune defense) and nephrocytes (mobile cells of the secretory system) are filled with unicellular stages of *P. canceri* (Thrupp et al. 2013). As other ascetosporean parasites show similar invasion patterns (Mourton et al. 1992; Stentiford et al. 2004; Elgharsalli et al. 2013), we hypothesize that *P. canceri* might also employ apoptotic mimicry to invade the host.

In conclusion, we used bioinformatic predictions and heterologous gene expression to investigate the energy and mitochondrial metabolisms in an emerging pathogen of animals (Hartikainen et al. 2014). Intracellular parasites like *P. canceri* are inherently difficult to study because they cannot be propagated in the lab and live in tight association with host tissue. We show that informative metabolic reconstruction of the parasite can be achieved from metagenomic data obtained from infected tissue, following a rigorous pipeline of *in silico* data sorting in combination with subcellular visualization of GFP fusion proteins heterologously expressed in yeast. This approach suggests a mitosome in *P. canceri* with a combination of features previously not described in other organisms with similar organelles. Importantly, our study also shows that despite the highly divergent nature of *P. canceri* proteins and mitosomal protein import machinery in general (no import machinery was found), *P. canceri* mitosomal proteins retain subcellular localization signals comparable to unrelated model organisms. Together, these methods represent promising avenues to further explore the biology of important yet poorly known uncultured parasites from natural host populations.

## Material & Methods

### Samples Collection and Pathological Investigation

Healthy and diseased individuals of juvenile edible crab (*Cancer pagurus*) were collected from Newton's Cove in Weymouth, UK, on June 26, 2014. Crabs were dissected and those individuals showing the characteristic signs of *P. canceri* infections (a proliferated antennal gland presenting as yellow gelatinous tissue) were classified as diseased candidates. Infections with *P. canceri* were further confirmed by histology following the protocol from Hartikainen et al. 2014 (Hartikainen et al. 2014) (supplementary fig. S1, Supplementary Material online).

### Nucleic Acid Extraction, Library Preparation and Sequencing

Nucleic acids were extracted from a highly infected antennal gland of a juvenile *C. pagurus* (diseased tissue) and a claw muscle of a healthy *C. pagurus* (healthy tissue) (supplementary fig. S1, Supplementary Material online). Briefly, tissues were homogenized using a Fast prep FP120 machine (MP biomedicals) at the highest setting for 2 min followed by an overnight incubation at 56 °C. The samples were subjected to centrifugation at 9000 rpm for 2 min and 50 *µ*l of the supernatant from each sample was added to 150 *µ*l of G2 buffer of the Qiagen DNeasy Blood and tissue kit. Total DNA was extracted from the homogenate using the EZ1 DNA tissue kit (cat no 953034) and the EZ1 advanced Biorobot (Qiagen) following manufacturer's instructions. For RNA extraction, frozen tissue samples were washed three times in PBS (Phosphate-buffered saline; 10 mM) by serial centrifugation. Complementary DNA (cDNA) was synthesized using the SMARTer Stranded Total RNA-Seq Kit v2—Pico Input Mammalian (Takara Bio) following the manual instructions.

Paired-end TrueSeq DNA libraries (2 × 100 bp) were prepared and sequenced with Illumina HiSeq2000. Illumina Nextera XT cDNA libraries (2 × 300 bp) were prepared and sequenced with Illumina MiSeq. The DNA libraries are referred to as: “healthy tissue DNA.HiSeq_2018” and “diseased tissue DNA.HiSeq_2018” and the cDNA libraries are referred to as: “healthy tissue cDNA.MiSeq_2018” and “diseased tissue cDNA.MiSeq_2018” (fig. 1, supplementary fig. S1 and Table S2, Supplementary Material online).

### Read Decontamination, Assemblies, and Quality Control

FastQC reports and assembly statistics before and after decontamination are shown in supplementary Table S2 and figure S1, Supplementary Material online. For all libraries the read quality and adaptor content was inspected with FASTQC v. 0.11.8 (Andrews 2010). Adaptor sequences were removed and low-quality reads were trimmed with Trimmomatic v. 0.36 (Bolger et al. 2014) under the following settings: ILLUMINACLIP:2:30:10 LEADING:10 TRAILING:10 SLIDINGWINDOW:4:20 MINLEN:30. The program KAT v.2.3.4 (Mapleson et al. 2017) was used to compare the k-mer frequencies and GC content of the two DNA libraries produced in this study.

To retrieve *P. canceri* reads from DNA and cDNA libraries of diseased crab tissue an iterative bioinformatic workflow was implemented (fig. 1). The workflow is divided into three parts accordingly: START 1: The host draft genome and transcriptome were assembled from the healthy tissue DNA and cDNA libraries using metaSpades v. 3.13.1 (k-mer auto option) (Bankevich et al. 2012) The reads of the diseased tissue DNA.HiSeq_2018 library were mapped on these assembly with BWA v.0.7.8 (Li and Durbin 2009). The unmapped reads were retrieved using Samtools v.1. 9 (Li et al. 2009) and assembled with SPAdes v. 3.13.1 (k-mer auto option) (Bankevich et al. 2012) resulting in a preliminary draft assembly of the diseased tissue (v. 1). The host and non-target contigs (e.g., metazoa, bacteria, fungi, plants) were identified based on megablast search against NCBI nucleotide database, BLAST v. 2.9.0+, visualized with Blobtools v.1.1.1 (Laetsch and Blaxter 2017) and removed based on Blobtools v.1.1.1 (Laetsch and Blaxter 2017) scripts, which resulted in a second draft assembly of the diseased tissue (v.2). The “diseased tissue DNA.HiSeq_2018 library” was re-mapped on the assembly v.2, the mapped reads were kept and re- assembled (Spades v. 3.13.1 (Bankevich et al. 2012), k = 87 option). The optimal k-mer threshold was determined with the KmerGenie v. 1.7039 (Chikhi and Medvedev 2014). The contigs of this third assembly (v.3) were queried using blastx searches (with Diamond v.0.9.29 (Buchfink et al. 2015)) against Diamond protein database (August 2020). All contigs that had hits against bacterial, archaeal, metazoan, Viridiplantae and fungal sequences with a bitscore and query coverage greater than 50% and 10%, respectively, were removed creating the *Pcanceri*_genome1. START 2: The read profiles of the DNA library generated in a previous study (Hartikainen et al. 2014) (noted as diseased tissue DNA.MiSeq_2014 library) were mapped on the first *P. canceri* assembly using BWA v.0.7.8 (Li and Durbin 2009). The mapped reads were retrieved Samtools v.1.9 (Li et al. 2009) normalized with BBmap v.38.08 (Bushnell 2014) at an average depth of minimum 5 × and maximum 100 × and assembled with SPAdes v. 3.13.1 (k-mer auto option) (Bankevich et al. 2012). This resulted in the second *P. canceri* genome assembly: *Pcanceri*_genome2. START 3: The cDNA reads of the diseased tissue library (diseased tissue cDNA.MiSeq_2018) were mapped with STAR v.2.7.2b (Dobin et al. 2013) on to the *Pcanceri*_genome1, the mapped reads were retrieved (Samtools v.1.9) and de novo assembled with Trinity v. 2.9.1 (Grabherr et al. 2011) in *P. canceri* transcriptome: Pcanceri_transcriptome. Additionally, the reads of the cDNA libraries obtained from the healthy and diseased tissue were de novo assembled with Trinity v. 2.9.1 in two metatranscriptomes.

To assess the levels of contamination within each assembly, the contigs were queried with Blast 2.9.0+ (Altschul et al. 1990) against the NCBI nucleotide (*nt*) database and the *P. canceri* reference genome to retrieve the top 500 hits per query below an e-value threshold of 1e-20. All contigs that had more than 10% hit coverage received a taxonomic identity using Blobtools v.1.1.1 (Laetsch and Blaxter 2017) (e.g., supplementary fig. S7, Supplementary Material online).

To assess the assembly size, contiguity and completeness statistics were generated with Quast v.4.5.4 (Alexey et al. 2013) for genomes, and TrinityStats.pl script of Trinity v. 2.9.1 (Grabherr et al. 2011) for transcriptomes. The assembly completeness was initially evaluated with BUSCO v. 3.0.2b (Simão et al. 2015) and Augustus v.3.2.3 (Stanke and Morgenstern 2005) searching both the nucleotide and protein sequences from the gene models, respectively, with the eukaryotes database (Table 1, supplementary fig. S2*A*, Supplementary Material online). BWA v.0.7.8 (Li and Durbin 2009) and Samtools v.1.9 (Li et al. 2009) were used to evaluate: 1) the percentage of reads that were included in the assembly and 2) the average read coverage for each assembly (estimated as number of mapped bases divided by the assembly size). The k-mer frequency plot of the *P.canceri_*genome1 assembly were constructed with KAT v2.3.4 (Mapleson et al. 2017). The intron aware alignment program STAR v.2.7.9a with StarLong algorithm (Dobin et al. 2013) was used to map the metatranscriptome of the diseased tissue onto the healthy tissue genome and transcriptome assemblies and on the *Pcanceri*_genome1 assembly. To further assign taxonomic identity to the unmapped transcripts, we used BLAST v. 2.12.0+ (Altschul et al. 1990); to identify homologues on NCBI nucleotides (blastx) and protein (blastp) databases (January 2022) using an e-value of 1e-50 and 1e-25, respectively. To identify *P. canceri* transcripts that did not map in the previous steps due to length difference, RNA editing, or synthetic chimeras, we performed additional blastn searches against the *Pcanceri*_genome1 assembly (e-value 1e-50). Based on transcript taxonomic identity, GC content and DNA read coverage we were able to separate *P. canceri* transcripts from host and other non-targeted organisms. These transcripts were mapped with STAR v.2.7.9a with StarLong algorithm on *Pcanceri*_genome1 assembly. As a last evaluation of the genome completeness, we used 127 genes retrieved from *M. mackini* transcriptome by previous studies (Burki et al. 2013; Schön et al. 2021). We used BLAST v. 2.12.0+ (Altschul et al. 1990) to search for homologues in the predicted proteomes of both *P. canceri* genomes.

### Gene Model Predictions, Functional Annotation, and Subcellular Localization

When comparing the open-reading frames in the transcriptomic data with their genomic counterparts, we failed to observe obvious spliceosomal introns. Therefore, Prodigal v. 2.6.3 (Hyatt et al. 2010), a bacterial gene prediction tool, was used to predict gene models and proteins for both *P. canceri* genome assemblies. TransDecoder v.5.3.0 (https://github.com/TransDecoder/TransDecoder) was used to identify candidate coding regions from all transcriptome assemblies generated in this study and the published transcriptome of *M. mackini* (Burki et al. 2013). Functional annotation of the predicted proteins was performed based on the following strategy. The predicted proteome was used as a query against the NCBI *nr* database (May 2020) to retrieve the top scoring hits (BLAST suite v. 2.9.0+). Interpro (IPR) domains were assigned using Interproscan v.5.30-69.0 (Jones et al. 2014). The online version of eggNOG-mapper v2 (Huerta-Cepas et al. 2017) was used for orthology assignments of the predicted proteins and K numbers were assigned on the GhostKoala web server (https://www.genome.jp/kegg/tool/map_pathway.html). The subcellular localization of each protein was determined with targetP v.2 (Almagro Armenteros et al. 2019) searching the non-plant organism group, MitoFates with fungal settings and DeepLoc-1.0 with default settings (Almagro Armenteros et al. 2017).

### Mitochondrial Protein Predictions and Downstream Analyses

Several searching strategies were employed to identify putative mitochondrial proteins in *P. canceri* and *M. mackini* (Burki et al. 2013). The predicted proteomes of *P. canceri* and *M. mackini* were inspected for the presence of proteins encoding MRO-localized proteins using the functional annotation and subcellular localization determined in the previous section. The output of eggNOG-mapper and Interproscan were additionally searched for any components of the protein import machinery or mitochondrial carrier family proteins. Moreover, the predicted mitochondrial proteomes from *Pygsuia biforma* (Stairs et al. 2014), *Blastocystis sp.* (Abrahamian et al. 2017) and *B. motovehiculus* (Gawryluk et al. 2016) were used as query sequences against the predicted proteins from *P. canceri* and *M. mackini* using BLAST v.2.1.8 (Altschul et al. 1990). Any protein that was predicted to be mitochondrial related based on at least one software tool used above or had one mitochondrial subject sequence retrieved in the top 100 BLAST hits was further investigated for completeness, annotation and mitosomal provenance. First, the gene model's completeness was assessed by manually examining the query coverage to similar sequences via BLAST. Those *P. canceri* predicted proteins that did not match to any sequence with BLAST or only aligned with hypothetical proteins were not examined but can be found in supplementary Table S1A and C, Supplementary Material online. Those predicted protein sequences with a methionine that align with the starting methionine of subject sequences were annotated as “complete“. In case that some but not all components of a certain metabolic pathway were predicted to be present in *P. canceri*, the putative missing genes were further searched in the metagenomic and metatranscriptomic predicted proteomes. If these searches proved negative, the raw reads of each library were further investigated with Phylomagnet (Schön et al. 2020). This program employs a gene centric approach to retrieve and assemble genes of interest directly from a raw read library. None of the investigated genes could be recovered from the raw reads.

### Phylogenetic Placement of *Paramikrocytos Canceri*

We constructed a phylogenomic dataset (supplementary fig. S3, Supplementary Material online) to confirm the taxonomic affiliation between *P. canceri* and *M. mackini*, based on data from previous work (Burki et al. 2013, Schön et al. 2021). In short, all (127) genes where a *M. mackini* ortholog was previously identified were used to search for homologs in the two *P. canceri* genomes by blastp to the protein predictions (e-value < 1e-15; query coverage > 50% as cutoff values). BLAST hits from *P. canceri* were aligned (MAFFT v. 7.310; E-INS-I) (Katoh and Standley 2013) with all sequences from Rhizaria retrieved from the full dataset compiled by Schön et al. (2021 -https://figshare.com/articles/dataset/Picozoa_cleaned_single_genes_initial_supermatrix_tree/14413712), excluding *Minchinia chitonis* due to poor gene representation. Stramenopiles, Alveolates and Telonemia from the taxon-reduced dataset in (Schön et al. 2021—https://figshare.com/articles/dataset/Picozoa_67_taxa_alignments_and_trees/14413592? file=27556019), were also included to provide outgroups to Rhizaria. In cases where the previous dataset contained several sequences from the same taxon, they were merged into OTUs using a custom script (https://github.com/markhilt/genome_analysis_tools/blob/master/merge_transcripts.py; v. 0.1). Single-gene trees were constructed using IQ-TREE2 v. 2.2.0.3 (using ModelFinder to determine the best-fit model of sequence evolution, restricting the search to site-homogeneous models with LG as base model) (Kalyaanamoorthy et al. 2017; Minh et al. 2020). Gene trees and multiple sequence alignments were visually inspected for potential paralogs and/or contaminants, where two such genes (*calm* and *rpl26*) were identified and excluded from the dataset. The remaining 125 alignments (all with *M. mackini* homologs, and 106 and 101 homologs in *P. canceri* genomes 1 and 2, respectively) were trimmed (trimAl v. 1.2, with the -gappyout parameter) and concatenated (https://github.com/SLAment/Genomics/blob/master/FastaManipulation/fastaconcat.py; v. 2.1; accessed 2022-02-15), resulting in a supermatrix of 40,962 amino acid sites with 55 taxa. The matrix was subjected to tree reconstruction in IQ-TREE2 v. 2.2.0.3 with 1,000 ultrafast bootstrap replicates to provide node support values (Minh et al. 2020). ModelFinder was used to determine the best-fit model, including the mixture models C20-60, where LG + C60 + F + I + G was selected. Unaligned amino acid sequences, aligned and trimmed sequences, and single-gene trees are available in FigShare repository (https://doi.org/10.6084/m9.figshare.21770576.v1).

### Phylogenetic Dataset Assembly

To confirm the taxonomic identity of the putative mitochondrial-related protein identified in *P. canceri* and eliminate the possibility of residual contamination, maximum-likelihood phylogenetic trees were constructed (supplementary fig. S5, Supplementary Material online). Except for the mitochondrial ABC transporter gene (*atm1*), the phylogenetic analysis workflow was performed as follows. All mitochondrial-related proteins identified in *P. canceri* were queried against the NCBI *nr* database (August, 2020) with BLAST v.2.1.9 (Altschul et al. 1990) using the BLASTP algorithm. The top 5,000 hits with an e-value less than 1e-10 (or 1e-5 if few hits were identified) were retrieved and clustered at 90% identity with CD-HIT v.4.8.1 (Edgar 2010). The predicted proteomes of *M. mackini* and *C. pagurus* were searched with BLASTP to retrieve homologous proteins. Lastly, a reciprocal BLASTP in all *P. canceri* predicted proteoms was performed. The sequences were aligned (Mafft v.7.407 (Katoh and Standley 2013), mafft-auto). The alignments were trimmed of ambiguous sites with (trimAL v.1.4.1 (Capella-Gutierrez et al. 2009), -automated1). The amino acid substitution model was determined with IQ-TREE2.1.6.5 using the default settings (Kalyaanamoorthy et al. 2017). Phylogenies and 1,000 ultrafast bootstrap trees with 1,000 SH-aLRT replicates were constructed with IQ-TREE2 v.1.6.5 (Minh et al. 2013). These initial phylogenies were visualized in FigTree v.1.4.4 and manually pruned to reduce the number of taxa. The reduced data set was aligned (Mafft v.7.407 (Katoh and Standley 2013), mafft-linsi). Removal of ambiguous sites, evaluation of amino acid substitution models, and phylogenetic reconstruction proceeded as above. For the putative *atm1* transporter, a Hidden Markov Model profile for orthologous group KOG0057 (retrieved from EggNOG 5.0.0 (Huerta-Cepas et al. 2019) database) was used to retrieve the protein models of *P. canceri* and *M. mackini* using the default settings of with hmmsearch. The resulting hits were used as queries against the NCBI nr database (August 2020) as described above. This dataset was supplemented with *atm1* sequences reported previously (Freibert et al. 2017). The proteins were aligned with hmmalign from HMMER v.3.2.1 (http://hmmer.org/) and the Atm1 phylogeny was performed as described above.

### Cloning of ISCU, mPGM, and PSS Genes

Full length *iscu, mpgm,* and *pss P. canceri* genes were commercially synthesized in pUC57 plasmids (Genewiz). The pDDGFP_LEUD plasmid (Addgene plasmid #58352) was used for expressing recombinant proteins in *Saccharomyces cerevisiae* (Parker and Newstead 2014). Plasmids containing genes of interest and the pDDGPD_LEU2D destination plasmid were recovered using the Nucleospin plasmid DNA purification kit (Macherey-Nagel) following the protocol provided by the manufacturer. Plasmids were linearized with 12 U/µl of SmaI (Promega) at 25 °C for 1 h. SmaI was inactivated at 70 °C for 15 min. Linearized plasmids were digested with 10 U/µl of BamHI (Promega) at 37 °C for 1 h and recipient pDDGPD_LEU2D was dephosphorylated with 1 µl of Thermosensitive Alkaline Phosphatase (TSAP, Promega) at 37 °C during the last 15 min of incubation with BamHI. Finally, TSAP and BamHI were inactivated at 74 °C for 15 min. Digestion products were ligated into dephosphorylated recipient plasmids at a 1:2 vector:insert ratio with 3 U of T4 DNA ligase (Promega) and at room temperature for 15 min according to the protocol of the manufacturer. Ligase was inactivated at 70 °C for 10 min. Exact protocols available upon request from the authors. *P. canceri* genes were cloned upstream of green fluorescent protein (*gfp*) encoded on the pDDGFP-LEU2D plasmid and transformed into *Escherichia coli* DH5α by standard methods and selected on synthetic defined media (LB plus 50 ug/ml ampicillin). Polymerase chain reactions (PCR) were performed to screen for colonies with gene insert using Gotaq Green master mix (Promega) and 10 µM of each primer (Forward primer: *gal1* 5′”-TCTGGGGTAATTAATCAGCGAAGCG-3′”. Reverse primers: *iscu* 5′”-GCTCCCGCAGCCGAAAGTCTTAAA −3′”, *pss* 5′”-AGGATGCGGCCTACTGAATGGACC-3′”, *mpgm* 5′”- GGGGCATAGGATCCATGACTCAATGGTGTGTA-3′” and *gfp* 5′”-AGTAGCGTCACCTTCACCTTCACC-3′”) using standard thermocycling conditions (95 °C 5 min; 30 cycles of 95 °C for 30 s, 55 °C for 30 s, 72 °C for 1 min, final elongation temperature of 72 °C for 10 min). The insertion of *P. canceri* genes into the pDDGFP_LEU2D vector was confirmed by Sanger sequencing with *gal1*_forward and *gfp*_reverse primers (Eurofins).

### Yeast Transformation and Heterologous Gene Expression

Wild-type yeast (W303-1A) were maintained in Synthetic Defined (SD; 0.067% yeast nitrogen base, 2% glucose, drop-out mix with or without uracil). pDDGFP-LEU2D-ISCU and pDDGFP-LEU2D-PSS were transformed into *S. cerevisiae* using the S. C. EasyComp Transformation Kit (Invitrogen) according to manufacturer”s protocol and selected on SD-ura; SD without uracil. For fluorescence microscopy, cells were grown on SD-ura + 2% glucose media overnight at 30 °C. A 100 µl aliquot of cells were transferred to SD-ura supplemented with 2% galactose and lacking glucose for the induction of the genes and incubated at 30 °C for 4–6 h. To stain mitochondria and nuclei, Mitotracker Red CMXRos (final concentration of 200 nM) was added to the cells for 20 min, followed by DAPI (2 µg/ml) for 10 min, respectively. After incubation, cells were washed with PBS and loaded in a 1% agarose pad. Cells were imaged on a Zeiss Axio Imager.z2 microscope, using a Plan-Apochromat 100×/1.40 Oil Ph 3 M27 immersion oil objective lens. Excitation wavelength: mCherry (Mitotracker) 587 nm, EGFP 488 nm, and DAPI 353 nm, emission wavelength: mCherry 610 nm, EGFP 509 nm, and DAPI 465 nm. Exposure times for each channel were: mCherry 100 ms, EGFP 500 ms, DAPI 170 ms. Images were processed using linear adjustments (e.g., brightness/contrast) and deconvolved using Zen. Expression of PSS resulted in changes to the yeast cell morphology; additional pictures are provided in supplementary figure S6, Supplementary Material online.

## Supplementary Material

evad022_Supplementary_Data

## Data Availability

Sequencing reads, and assemblies generated in this study are available the European Nucleotide Archive (ENA) under the study PRJEB46940 (https://www.ebi.ac.uk/ena/browser/view/PRJEB46940). The assemblies of *P. canceri* genome 1 and transcriptome together with the predicted proteomes are available at figshare (https://doi.org/10.6084/m9.figshare.21206669.v1). Phylogenetic datasets for the MRO genes and *P.canceri* phylogenetic placement are available at figshare (https://doi.org/10.6084/m9.figshare.21206807.v1 and https://doi.org/10.6084/m9.figshare.21770576.v1, respectively). The scripts used in this study are available at https://github.com/IoanaBrannstrom/Pcanceri_MRO.

## References

[evad022-B1] Abrahamian M , Kagda M, Ah-Fong AMV, Judelson HS. 2017. Rethinking the evolution of eukaryotic metabolism: novel cellular partitioning of enzymes in stramenopiles links serine biosynthesis to glycolysis in mitochondria. BMC Evol Biol. 17:241. 10.1186/s12862-017-1087-8 PMC5715807 29202688

[evad022-B2] Acoba Michelle Grace , Senoo Nanami, Claypool Steven M. 2020. Phospholipid ebb and flow makes mitochondria go. J Cell Biol. 219(8):525. 10.1083/jcb.202003131 PMC7401802 32614384

[evad022-B3] Alexey G , Vladislav S, Nikolay V, Glenn T. 2013. QUAST: quality assessment tool for genome assemblies. Bioinformatics 29(8):1072–1075. 23422339 10.1093/bioinformatics/btt086 PMC3624806

[evad022-B4] Almagro Armenteros JJ , et al 2017. Deeploc: prediction of protein subcellular localization using deep learning. Bioinformatics 33(21):3387–3395. 29036616 10.1093/bioinformatics/btx431

[evad022-B5] Almagro Armenteros JJ , et al 2019. Detecting sequence signals in targeting peptides using deep learning. Life Sci Alliance. 2(5):e201900429. 10.26508/lsa.201900429 PMC6769257 31570514

[evad022-B6] Altschul SF , Gish W, Miller W, Myers EW, Lipman DJ. 1990. Basic local alignment search tool. J Mol Biol. 215(3):403–410. 2231712 10.1016/S0022-2836(05)80360-2

[evad022-B7] Amara A , Mercer J. 2015. Viral apoptotic mimicry. Nat Rev Microbiol. 13(8):461–469. 26052667 10.1038/nrmicro3469 PMC7097103

[evad022-B8] Andrews S. 2010. FastQC: a quality control tool for high throughput sequence data [Online]. http://www.bioinformatics.babraham.ac.uk/projects/fastqc.

[evad022-B9] Bankevich A , et al 2012. SPAdes: a new genome assembly algorithm and its applications to single-cell sequencing. J Comput Biol. 19(5):455–477. 22506599 10.1089/cmb.2012.0021 PMC3342519

[evad022-B10] Bass D , Ward MG, Burki F. 2019. Ascetosporea. Curr Biol. 29(1):R7–R8. 30620917 10.1016/j.cub.2018.11.025

[evad022-B11] Bateman KS , Hicks RJ, Stentiford GD. 2011. Disease profiles differ between non-fished and fished populations of edible crab (*Cancer pagurus*) from a major commercial fishery. ICES J Marine Sci. 68(10):2044–2052.

[evad022-B12] Biard T . 2023. Rhizaria. In *eLS*, John Wiley & Sons, Ltd (Ed.).

[evad022-B13] Bolger AM , Lohse M, Usadel B. 2014. Trimmomatic: a flexible trimmer for illumina sequence data. Bioinformatics 30(15):2114–2120. 24695404 10.1093/bioinformatics/btu170 PMC4103590

[evad022-B14] Buchfink B , Xie C, Huson DH. 2015. Fast and sensitive protein alignment using DIAMOND. Nat Methods. 12(1):59–60. 25402007 10.1038/nmeth.3176

[evad022-B15] Burki F , et al 2013. Phylogenomics of the Intracellular Parasite *Mikrocytos mackini* Reveals Evidence for a Mitosome in Rhizaria. Curr Biol. 23(16):1541–1547. 23891116 10.1016/j.cub.2013.06.033

[evad022-B16] Burki F , Keeling PJ. 2014. Rhizaria. Curr Biol. 24(3):R103–R107. 24502779 10.1016/j.cub.2013.12.025

[evad022-B17] Bushnell B . 2014. BBMap: A Fast, Accurate, Splice-Aware Aligner. *United States: N. p. Web*.

[evad022-B18] Calianese DC , Birge RB. 2020. Biology of phosphatidylserine (PS): basic physiology and implications in immunology, infectious disease, and cancer. Cell Commun Signal. 18(1):41. 32160904 10.1186/s12964-020-00543-8 PMC7065380

[evad022-B19] Calzada E , et al 2019. Phosphatidylethanolamine made in the inner mitochondrial membrane is essential for yeast cytochrome bc1 complex function. Nat Commun. 10(1):997. 30926815 10.1038/s41467-019-09425-1 PMC6441012

[evad022-B20] Campbell SE , et al 2013. The genome of *Spraguea lophii* and the basis of host-microsporidian interactions. PLoS Genet. 9(8):e1003676. 10.1371/journal.pgen.1003676 PMC3749934 23990793

[evad022-B21] Capella-Gutierrez S , Silla-Martinez J-M, Galbadon T. 2009. Trimal: a tool for automated alignment trimming in large-scale phylogenetic analyses. Bioinformatics 25:1972–1973. 19505945 10.1093/bioinformatics/btp348 PMC2712344

[evad022-B22] Carman MG , Han GS. 2011. Regulation of phospholipid synthesis in the yeast *Saccharomyces cerevisiae*. Annu Rev Biochem. 80:859–883. 10.1146/annurev-biochem-060409-092229 PMC3565220 21275641

[evad022-B23] Chan KW , et al 2005. A novel ADP/ATP transporter in the mitosome of the microaerophilic human parasite *Entamoeba histolytica*. Curr Biol. 15(8):737–742. 15854906 10.1016/j.cub.2005.02.068

[evad022-B24] Chikhi R , Medvedev P. 2014. Informed and automated k-mer size selection for genome assembly. Bioinformatics 30(1):31–37. 23732276 10.1093/bioinformatics/btt310

[evad022-B25] Dobin A , et al 2013. STAR: ultrafast universal RNA-seq aligner. Bioinformatics 29(1):15–21. 23104886 10.1093/bioinformatics/bts635 PMC3530905

[evad022-B26] Edgar RC . 2010. Search and clustering orders of magnitude faster than BLAST. Bioinformatics 26(19):2460–2461. 20709691 10.1093/bioinformatics/btq461

[evad022-B27] El-Hani CN , et al 2012. Apoptosis and apoptotic mimicry in *Leishmania*: an evolutionary perspective. Front Cell Infect Microbiol. 2:1–8. 22912937 10.3389/fcimb.2012.00096 PMC3418608

[evad022-B28] El Alaoui H , et al 2001. *Encephalitozoon cuniculi* (Microspora): characterization of a phospholipid metabolic pathway potentially linked to therapeutics. Exp Parasitol. 98(4):171–179. 11560410 10.1006/expr.2001.4635

[evad022-B29] Elgharsalli R , et al 2013. Characterization of the protozoan parasite marteilia refringens infecting the dwarf oyster ostrea stentina in Tunisia. J Invertebr Pathol. 112(2):175–183. 23219430 10.1016/j.jip.2012.11.004

[evad022-B30] Freibert S-A , et al 2017. Evolutionary conservation and in vitro reconstitution of microsporidian iron–sulfur cluster biosynthesis. Nat Commun. 8:1. 28051091 10.1038/ncomms13932 PMC5216125

[evad022-B31] Garcia C , et al 2018. Descriptions of *Mikrocytos veneroïdes* n. sp. and *Mikrocytos donaxi* n. sp. (Ascetosporea: Mikrocytida: Mikrocytiidae), detected during important mortality events of the wedge clam *Donax trunculus* linnaeus (Veneroida: Donacidae), in France between 2008 and 2011. Parasit Vectors 11:119. 29499746 10.1186/s13071-018-2692-0 PMC5834847

[evad022-B32] Gawryluk RMR , et al 2016. The earliest stages of mitochondrial adaptation to low oxygen revealed in a novel rhizarian. Curr Biol. 26(20):2729–2738. 27666965 10.1016/j.cub.2016.08.025

[evad022-B33] Gawryluk RMR , Stairs CW. 2021. Diversity of electron transport chains in anaerobic protists. Biochim Biophys Acta Bioenerg. 1862:148334. 33159845 10.1016/j.bbabio.2020.148334

[evad022-B34] Glancy B , et al 2020. Mitochondrial lactate metabolism: history and implications for exercise and disease. J Physiol. 599(3):863–888. 32358865 10.1113/JP278930 PMC8439166

[evad022-B35] Goldberg AV , et al 2008. Localization and functionality of microsporidian iron–sulphur cluster assembly proteins. Nature 452(7187):624–628. 18311129 10.1038/nature06606

[evad022-B36] Grabherr MG , et al 2011. Full-length transcriptome assembly from RNA-seq data without a reference genome. Nat Biotechnol. 29(7):644–652. 21572440 10.1038/nbt.1883 PMC3571712

[evad022-B37] Hartikainen H , et al 2013. Lineage-specific molecular probing reveals novel diversity and ecological partitioning of haplosporidians. ISME J. 8(1):177–186. 23966100 10.1038/ismej.2013.136 PMC3869015

[evad022-B38] Hartikainen H , et al 2014. Mikrocytids are a broadly distributed and divergent radiation of parasites in aquatic invertebrates. Curr Biol. 24(7):807–812. 24656829 10.1016/j.cub.2014.02.033

[evad022-B39] Huerta-Cepas J , et al 2017. Fast genome-wide functional annotation through orthology assignment by eggNOG-mapper. Mol Biol Evol. 34(8):2115–2122. 28460117 10.1093/molbev/msx148 PMC5850834

[evad022-B40] Huerta-Cepas J , et al 2019. eggNOG 5.0: a hierarchical, functionally and phylogenetically annotated orthology resource based on 5090 organisms and 2502 viruses. Nucleic Acids Res. 47(D1):D309–D314. 30418610 10.1093/nar/gky1085 PMC6324079

[evad022-B41] Hyatt D , et al 2010. Prodigal: prokaryotic gene recognition and translation initiation site identification. BMC Bioinformatics 11(1):119. 20211023 10.1186/1471-2105-11-119 PMC2848648

[evad022-B42] Jedelský PL , et al 2011. The minimal proteome in the reduced mitochondrion of the parasitic protist *Giardia intestinalis*. PLoS One. 6(2):e17285. 21390322 10.1371/journal.pone.0017285 PMC3044749

[evad022-B43] Johnson PT . 1987. A review of fixed phagocytic and pinocytotic cells of decapod crustaceans, with remarks on hemocytes. Dev Comp Immunol. 11:679–704. 3326757 10.1016/0145-305x(87)90057-7

[evad022-B44] Jones P , et al 2014. Interproscan 5: genome-scale protein function classification. Bioinformatics (Oxford, England) 30(9):1236–1240. 24451626 10.1093/bioinformatics/btu031 PMC3998142

[evad022-B45] Kalyaanamoorthy S , et al 2017. Modelfinder: fast model selection for accurate phylogenetic estimates. Nat Methods. 14(6):587–589. 28481363 10.1038/nmeth.4285 PMC5453245

[evad022-B46] Karnkowska A , et al 2016. A eukaryote without a mitochondrial organelle. Curr Biol. 26(10):1274–1284. 27185558 10.1016/j.cub.2016.03.053

[evad022-B47] Katinka MD , et al 2001. Genome sequence and gene compaction of the eukaryote parasite *Encephalitozoon cuniculi*. Nature 414(6862):450–453. 11719806 10.1038/35106579

[evad022-B48] Katoh K , Standley DM. 2013. MAFFT Multiple sequence alignment software version 7: improvements in performance and usability. Mol Biol Evol. 30:772–780. 23329690 10.1093/molbev/mst010 PMC3603318

[evad022-B49] Kay JG , Fairn GD. 2019. Distribution, dynamics and functional roles of phosphatidylserine within the cell. Cell Commun Signaling 17(1):12888. 10.1186/s12964-019-0438-z PMC6792266 31615534

[evad022-B50] Laetsch DR , Blaxter ML. 2017. Blobtools: interrogation of genome assemblies [version 1; peer review: 2 approved with reservations]. F1000Res. 6:1287.

[evad022-B51] Leger MM , et al 2016. Novel hydrogenosomes in the microaerophilic jakobid *Stygiella incarcerata*. Mol Biol Evol. 33(9):2318–2336. 27280585 10.1093/molbev/msw103 PMC4989108

[evad022-B53] Li H , Durbin R. 2009. Fast and accurate short read alignment with burrows–wheeler transform. Bioinformatics 25(14):1754–1760. 19451168 10.1093/bioinformatics/btp324 PMC2705234

[evad022-B52] Li H , et al 2009. The sequence alignment/map format and SAMtools. Bioinformatics 25(16):2078–2079. 19505943 10.1093/bioinformatics/btp352 PMC2723002

[evad022-B54] Liaud M-F , et al 2000. Compartment-Specific isoforms of TPI and GAPDH are imported into diatom mitochondria as a fusion protein: evidence in favor of a mitochondrial origin of the eukaryotic glycolytic pathway. Mol Biol Evol. 17(2):213–223. 10677844 10.1093/oxfordjournals.molbev.a026301

[evad022-B55] Mai Z , et al 1999. Hsp60 is targeted to a cryptic mitochondrion-derived organelle (“crypton”) in the microaerophilic protozoan parasite *Entamoeba histolytica*. Mol.Cell Biol. 19(3):2198–2205. 10022906 10.1128/mcb.19.3.2198 PMC84012

[evad022-B56] Makiuchi T , Nozaki T. 2014. Highly divergent mitochondrion-related organelles in anaerobic parasitic protozoa. Biochimie 100:3–17. 24316280 10.1016/j.biochi.2013.11.018

[evad022-B57] Mapleson D , et al 2017. KAT: a K-mer analysis toolkit to quality control NGS datasets and genome assemblies. Bioinformatics (Oxford, England) 33(4):574–576. 27797770 10.1093/bioinformatics/btw663 PMC5408915

[evad022-B58] Mathur V , Wakeman KC, Keeling PJ. 2021. Parallel functional reduction in the mitochondria of apicomplexan parasites. Curr Biol. 31(13):2920–2928 33974849 10.1016/j.cub.2021.04.028

[evad022-B59] Mi-ichi F , et al 2009. Mitosomes in *Entamoeba histolytica* contain a sulfate activation pathway. Proc Natal Acad Sci U S A. 106(51):21731–21736. 10.1073/pnas.0907106106 PMC2799805 19995967

[evad022-B61] Minh BQ , Nguyen MAT, von Haeseler A. 2013. Ultrafast approximation for phylogenetic bootstrap. Mol Biol Evol. 30(5):1188–1195. 23418397 10.1093/molbev/mst024 PMC3670741

[evad022-B60] Minh BQ , et al 2020. IQ-TREE 2: new models and efficient methods for phylogenetic inference in the genomic era. Mol Biol Evol. 37:1530–1534. 32011700 10.1093/molbev/msaa015 PMC7182206

[evad022-B62] Mourton C , et al 1992. Interactions between *Bonamia ostreae* (protozoa: ascetospora) and hemocytes of *Ostrea edulis* and *Crassostrea gigas* (Mollusca: bivalvia): in vitro system establishment. J Invertebr Pathol. 59:235–240.

[evad022-B63] Müller M . 1993. The hydrogenosome. Microbiology 139(12):2879–2889. 10.1099/00221287-139-12-2879 8126416

[evad022-B64] Müller M , et al 2012. Biochemistry and evolution of anaerobic energy metabolism in eukaryotes. Microbiol Mol Biol Rev. 76(2):444–495. 22688819 10.1128/MMBR.05024-11 PMC3372258

[evad022-B65] Nakayama T , Ishida K-I, Archibald JM. 2012. Broad distribution of TPI-GAPDH fusion proteins among eukaryotes: evidence for glycolytic reactions in the mitochondrion? PLoS One 7(12):e52340. 23284996 10.1371/journal.pone.0052340 PMC3527533

[evad022-B66] Nývltová E , et al 2015. Lateral gene transfer and gene duplication played a key role in the evolution of *Mastigamoeba balamuthi* hydrogenosomes. Mol Biol Evol. 32(4):1039–1055. 25573905 10.1093/molbev/msu408 PMC4379409

[evad022-B67] Parker JL , Newstead S. 2014. Method to increase the yield of eukaryotic membrane protein expression in *Saccharomyces cerevisiae* for structural and functional studies. Protein Sci. 23(9):1309–1314. 24947543 10.1002/pro.2507 PMC4230410

[evad022-B68] Ramakrishnan S , et al 2013. Lipid synthesis in protozoan parasites: a comparison between kinetoplastids and apicomplexans. Prog Lipid Res. 52(4):488–512. 23827884 10.1016/j.plipres.2013.06.003 PMC3830643

[evad022-B69] Río Bártulos C , et al 2018. Mitochondrial glycolysis in a Major lineage of eukaryotes. Genome Biol Evol. 10(9):2310–2325. 30060189 10.1093/gbe/evy164 PMC6198282

[evad022-B70] Roger AJ , Muñoz-Gómez SA, Kamikawa R. 2017. The origin and diversification of mitochondria. Curr Biol. 27(21):R1177–R1192. 29112874 10.1016/j.cub.2017.09.015

[evad022-B71] Salomaki ED , et al 2021. Gregarine single-cell transcriptomics reveals differential mitochondrial remodeling and adaptation in apicomplexans. BMC Biol. 19(1):77. 33863338 10.1186/s12915-021-01007-2 PMC8051059

[evad022-B72] Santos HJ , Makiuchi T, Nozaki T. 2018. Reinventing an organelle: the reduced mitochondrion in parasitic protists. Trends Parasitol. 34(12):1038–1055. 30201278 10.1016/j.pt.2018.08.008

[evad022-B74] Schön ME , Eme L, Ettema TJG. 2020. Phylomagnet: fast and accurate screening of short-read meta-omics data using gene-centric phylogenetics. Bioinformatics 36(6):1718–1724. 31647547 10.1093/bioinformatics/btz799 PMC7703773

[evad022-B73] Schön ME , et al 2021. Single cell genomics reveals plastid-lacking picozoa are close relatives of red algae. Nat Commun. 12:1. 34789758 10.1038/s41467-021-26918-0 PMC8599508

[evad022-B79] Stairs CW , Leger MM, Roger AJ. 2015. Diversity and origins of anaerobic metabolism in mitochondria and related organelles. Phil Trans R Soc B 370:20140326. 26323757 10.1098/rstb.2014.0326 PMC4571565

[evad022-B75] Simão FA , et al 2015. BUSCO: assessing genome assembly and annotation completeness with single-copy orthologs. Bioinformatics 31(19):3210–3212. 26059717 10.1093/bioinformatics/btv351

[evad022-B76] Smith DGS , et al 2007. Exploring the mitochondrial proteome of the Ciliate protozoon *Tetrahymena thermophila*: direct analysis by tandem mass spectrometry. J Mol Biol. 374(3):837–863. 17959197 10.1016/j.jmb.2007.09.051

[evad022-B77] Stairs CW , et al 2014. A SUF Fe-S cluster biogenesis system in the mitochondrion-related organelles of the anaerobic protist *Pygsuia*. Curr Biol. 24(11):1176–1186. 24856215 10.1016/j.cub.2014.04.033

[evad022-B78] Stairs CW , et al 2021. Anaeramoebae are a divergent lineage of eukaryotes that shed light on the transition from anaerobic mitochondria to hydrogenosomes. Curr Biol. 31(24):5605–5612.e5605. 34710348 10.1016/j.cub.2021.10.010

[evad022-B80] Stanke M , Morgenstern B. 2005. AUGUSTUS: a web server for gene prediction in eukaryotes that allows user-defined constraints. Nucleic Acids Res. 33(Web Server issue):W465–W467. 15980513 10.1093/nar/gki458 PMC1160219

[evad022-B81] Stentiford GD , Feist SW, Bateman KS. 2004. Haemolymph parasite of the shore crab *Carcinus maenas*: pathology, ultrastructure and observations on crustacean haplosporidians. Dis Aquatic Organ. 59:57–68. 10.3354/dao059057 15212293

[evad022-B82] Thrupp TJ , et al 2013. Infection of juvenile edible crabs, *Cancer pagurus* by a haplosporidian-like parasite. J Invertebr Pathol. 114(1):92–99. 23796496 10.1016/j.jip.2013.06.003

[evad022-B83] Tovar J , et al 2003. Mitochondrial remnant organelles of *Giardia* function in iron-sulphur protein maturation. Nature 426(13):172–176. 14614504 10.1038/nature01945

[evad022-B84] Tsaousis AD , et al 2008. A novel route for ATP acquisition by the remnant mitochondria of *Encephalitozoon cuniculi*. Nature 453(7194):553–556. 18449191 10.1038/nature06903

[evad022-B85] Vance J , Steenbergen R. 2005. Metabolism and functions of phosphatidylserine. Prog Lipid Res. 44(4):207–234. 15979148 10.1016/j.plipres.2005.05.001

[evad022-B86] Van der Giezen M , Tovar J. 2005. Degenerate mitochondria. EMBO Rep. 6(6):525–530. 15940286 10.1038/sj.embor.7400440 PMC1369098

[evad022-B87] Vial HJ , et al 2003. Phospholipids in parasitic protozoa. Mol Biochem Parasitol. 126(2):143–154. 12615313 10.1016/s0166-6851(02)00281-5

[evad022-B88] Wanderley JLM , DaMatta RA, Barcinski MA. 2020. Apoptotic mimicry as a strategy for the establishment of parasitic infections: parasite- and host-derived phosphatidylserine as key molecule. Cell Commun Signal. 18:10. 31941500 10.1186/s12964-019-0482-8 PMC6964003

[evad022-B89] Yichoy M , et al 2010. Lipid metabolism in *Giardia*: a post-genomic perspective. Parasitology 138(3):267–278. 20880419 10.1017/S0031182010001277 PMC3132189

[evad022-B90] Zítek J , et al 2022 . Reduced mitochondria provide an essential function for the cytosolic methionine cycle. Curr Biol. 32(23):5057–5068.e5. 36347252 10.1016/j.cub.2022.10.028 PMC9746703

