# Supplementary material for: A Mitosome With Distinct Metabolism in the Uncultured Protist Parasite Paramikrocytos canceri (Rhizaria, Ascetosporea)
Source: Genome Biol Evol. 2023 Feb 15;15(3):evad022. doi: 10.1093/gbe/evad022 (PMC9998036; doi:10.1093/gbe/evad022)
Supplement: evad022_Supplementary_Data [file evad022_supplementary_data.zip › Suppl-data_GBE_revision_FINAL_corrected.pdf]

A mitosome with distinct metabolism in the uncultured protist parasite *Paramikrocytos canceri* (Rhizaria, Ascetosporea)

Supplemental data

Fig. S1. Experimental setup.
(A) Upon dissection (**1**), the diseased individual showed clear signs of heavy infection with *P. canceri* based on the proliferated antennal gland: see the yellow gelatinous tissue pinpointed with a black arrow. *P.canceri* infections were further confirmed by histology (**2**). The blue arrow indicates the cell wall of the host cell. White arrows show *P. canceri* cells with single and multinucleate nuclei, while yellow arrows show the plasmodial stage of the parasite. The sections and histological work was performed as in Hartikainen *et al* 2014. **(B)** The figure depicts the methodological approach to obtain nucleic acids from highly infected host antennal gland and healthy muscle of the crab claw. Illumina TrueSeq P100 libraries were used for the DNA isolated from both healthy and diseased tissue. The two DNA libraries were pooled together and sequenced on one HiSeq2000 Illumina lane. RNA was isolated from the same tissues (healthy and diseased) and transformed in cDNA, which was further transformed in two Illumina Nextera XT PE300 libraries. The libraries were pooled together and sequenced on one MiSeq lane.

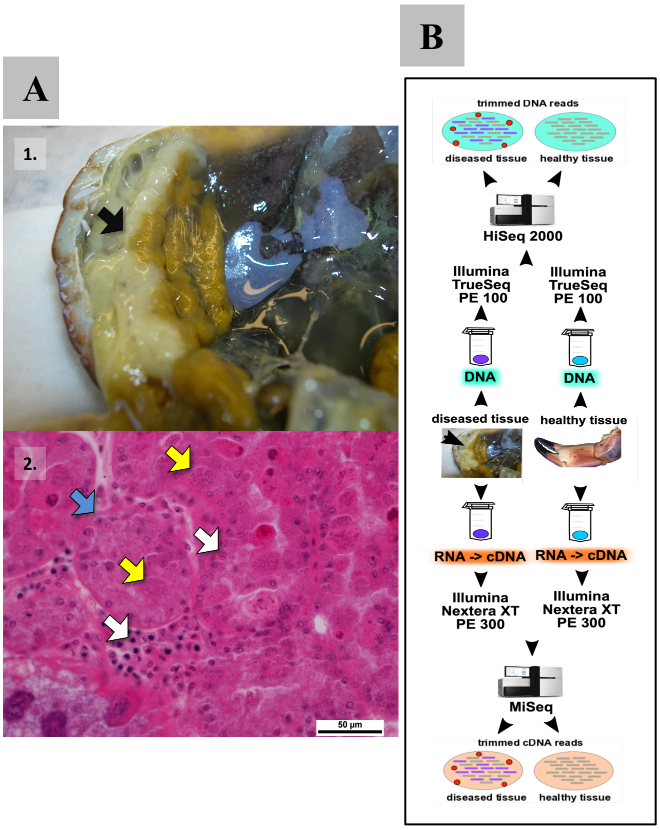

Fig.S2: (A) Busco % values for each genome assembly (nucleotide level).
The Busco values are based on eukaryote_odb9 database. **(B)**. K-mer frequency plot for the P.canceri genome1 assembly and its respective library reads

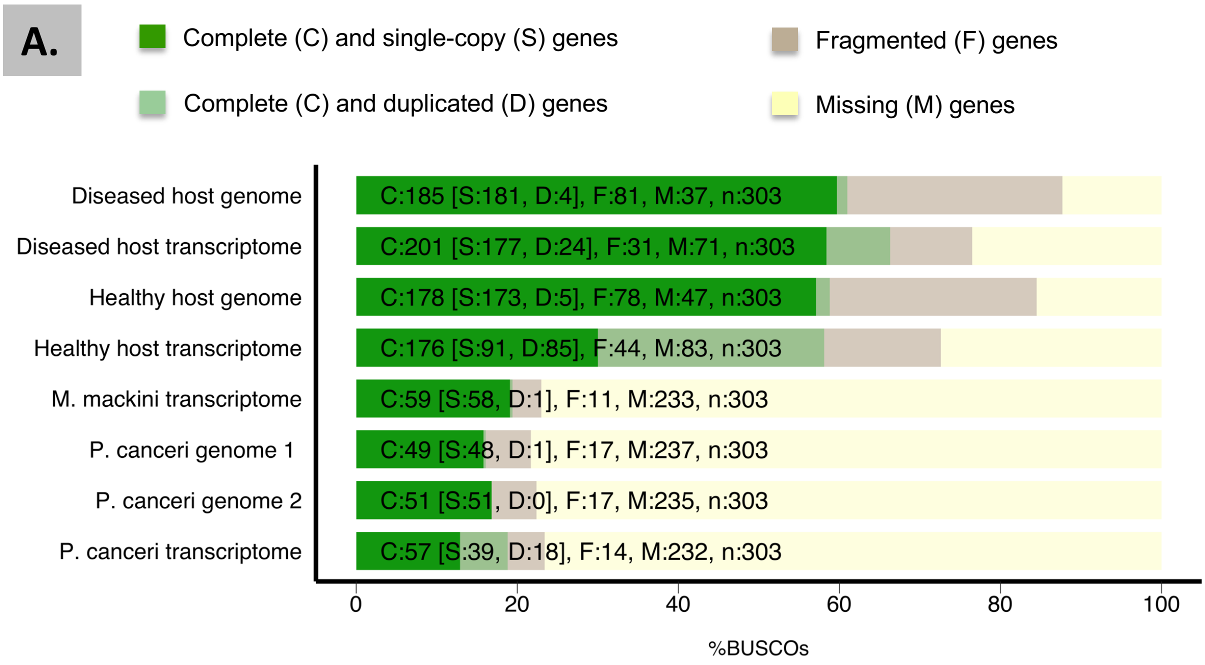

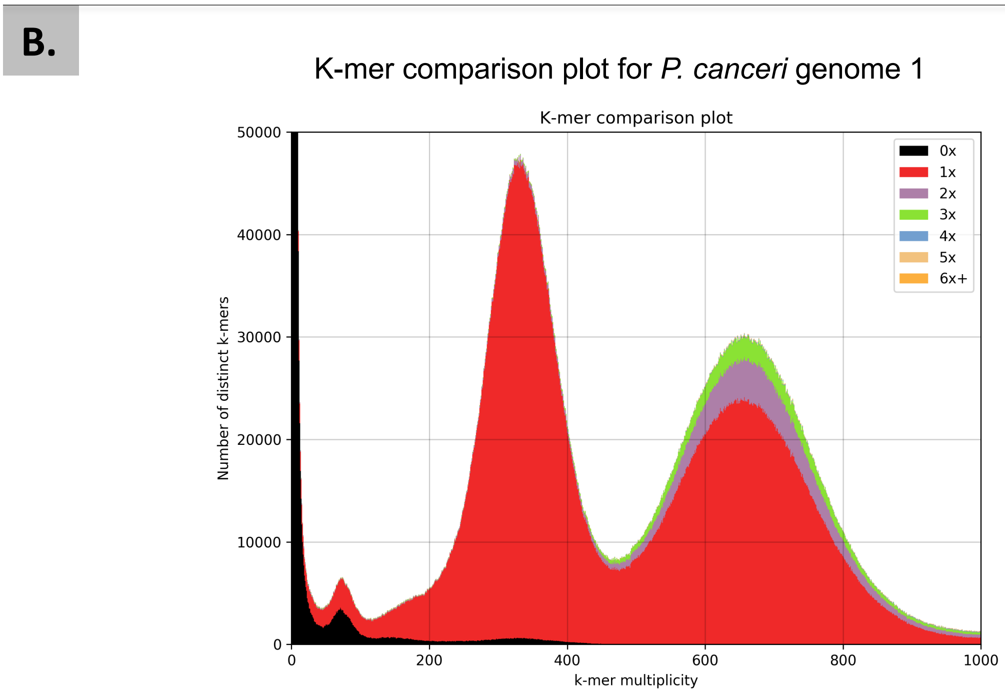

Fig. S3. Phylogenetic placement of *P. canceri* and *M. mackini*

IQ –TREE2 inferred with 125 orthologues under the LG+C60+F+I+G model, as implemented in IQtree. Values at nodes denote maximum likelihood bootstrap support. The scale bar represents the estimated number of amino acid substitutions per site. Stramenopila, Alveolata and Telonemia were used as outgroups.

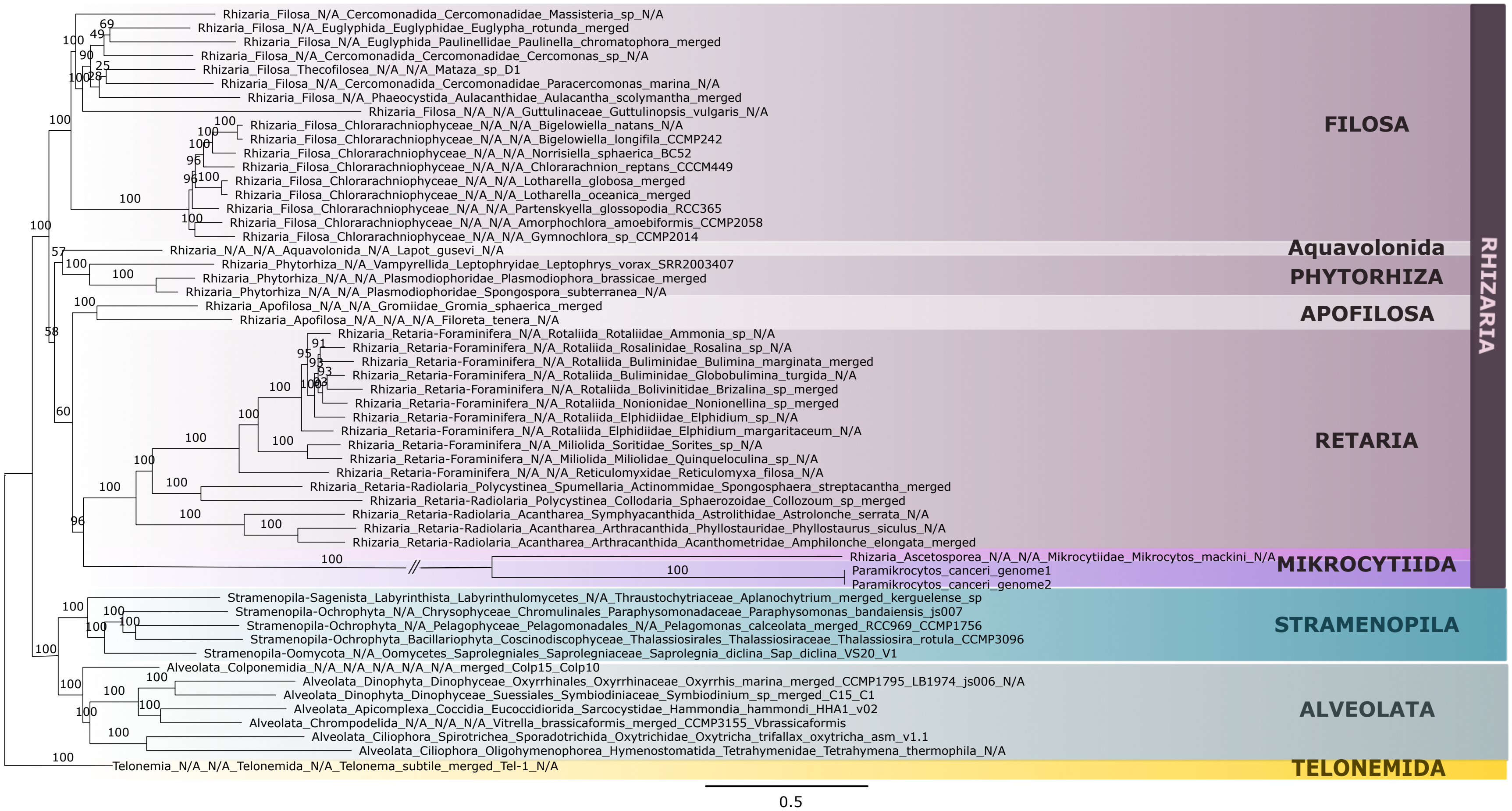

Fig. S4. A. Alignament of *P. canceri* mLDH (mitochondrial lactate dehydrogenase). Only the first 117 amino acids are shown.

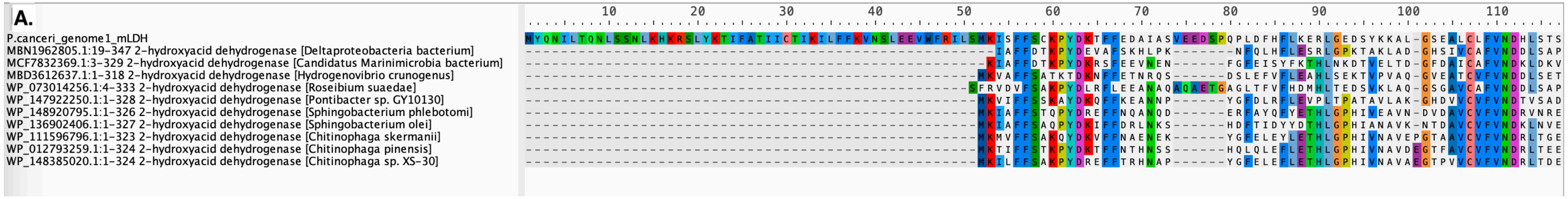

Fig. S4. B. Scaffolds containing the mLDH gene in P. canceri genome 1 and P. canceri genome 2**.** The scaffolds, their read coverage and the gene models (annotated by Prodigal) were visualized in IGV. The red lines show the mate-pair coverage support for the scaffold continuity.

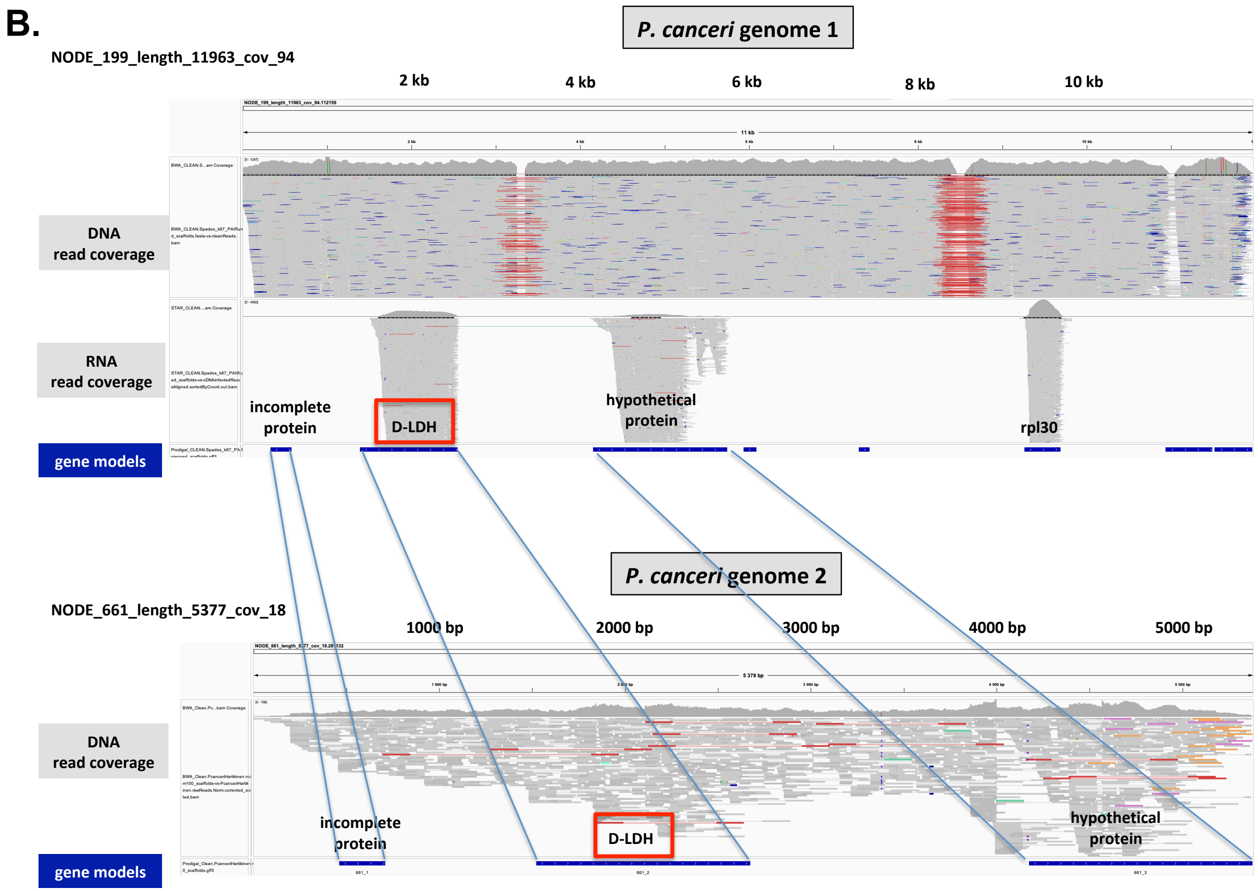

Fig. S5. Phylogenetic relationships of *P. canceri* and *M. mackini* genes involved in the glycolytic, CDP-DAG, ISC pathway for Fe-S cluster biosynthesis and protein folding pathways.

The parameters to align and trim the alignments together with amino acid substitution model and the taxa used to root the trees are shown in the figure legend. The fasta files used for all the phylogenetic analysis and the corresponding tree files are deposited at https://figshare.com/s/9041cf9ba46dfad931c8

Fig. S5. Glycolysis pathway - preparatory phase – Step 1: GCK (glucokinase)

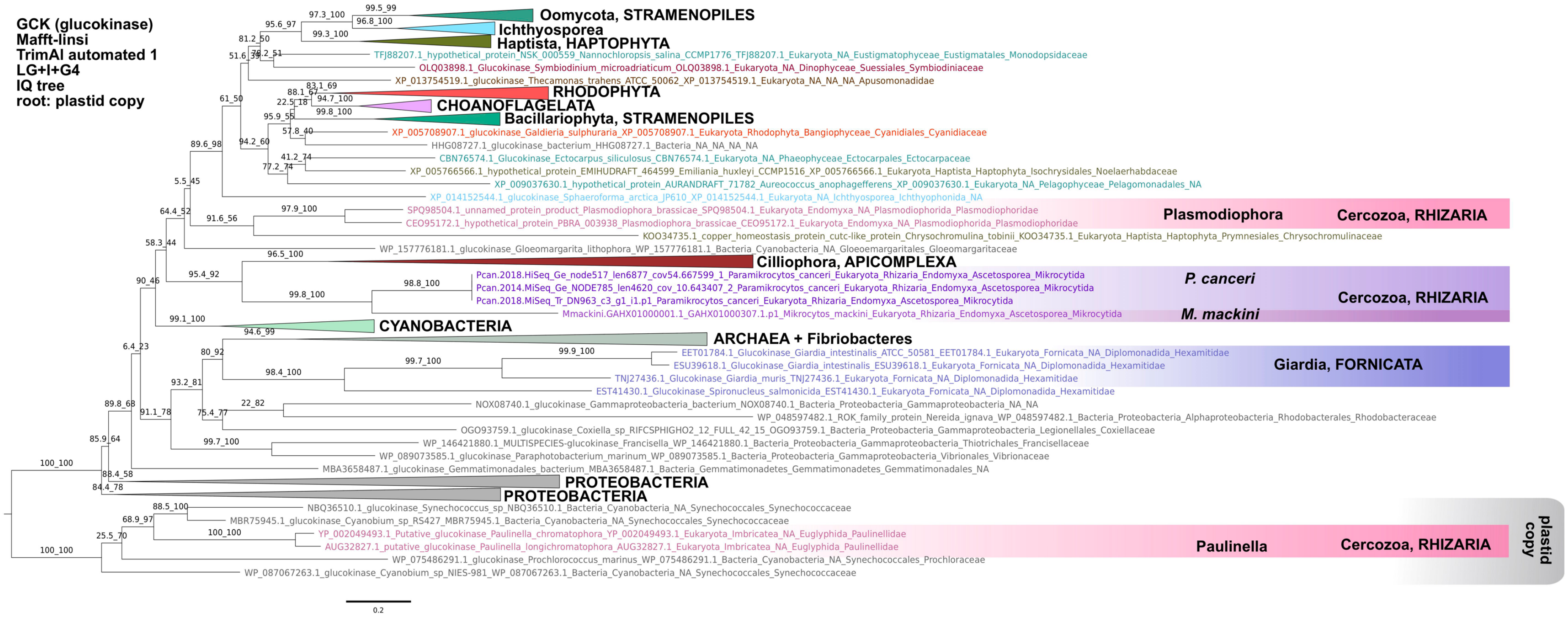

Fig. S5. Glycolysis pathway - preparatory phase – Step 2: GPI (glucose-3-phosphate isomerize)

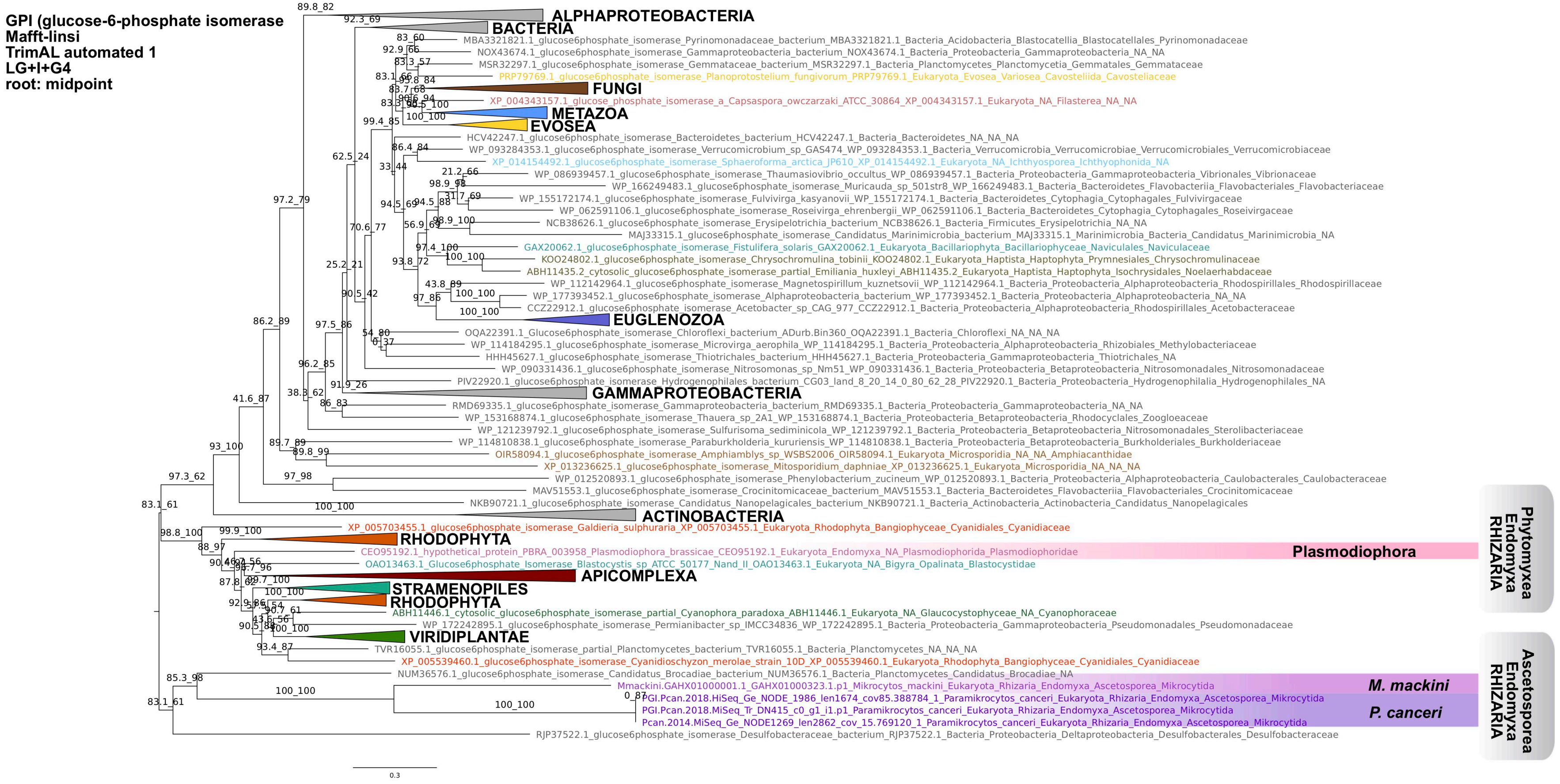

Fig. S5. Glycolysis pathway - preparatory phase – Step 3: PFK (phosphofructokinase)

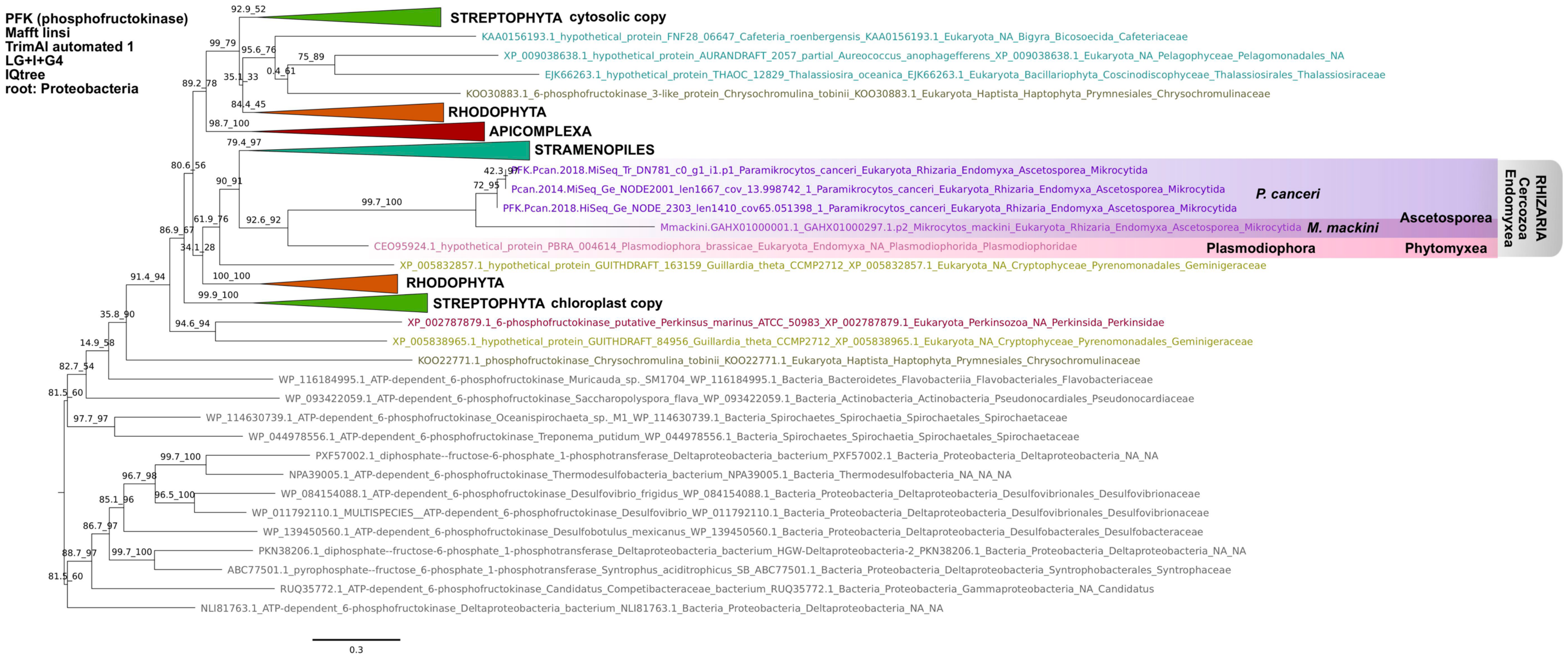

Fig. S5. Glycolysis pathway - preparatory phase – Step 4: ALDO (aldolase)

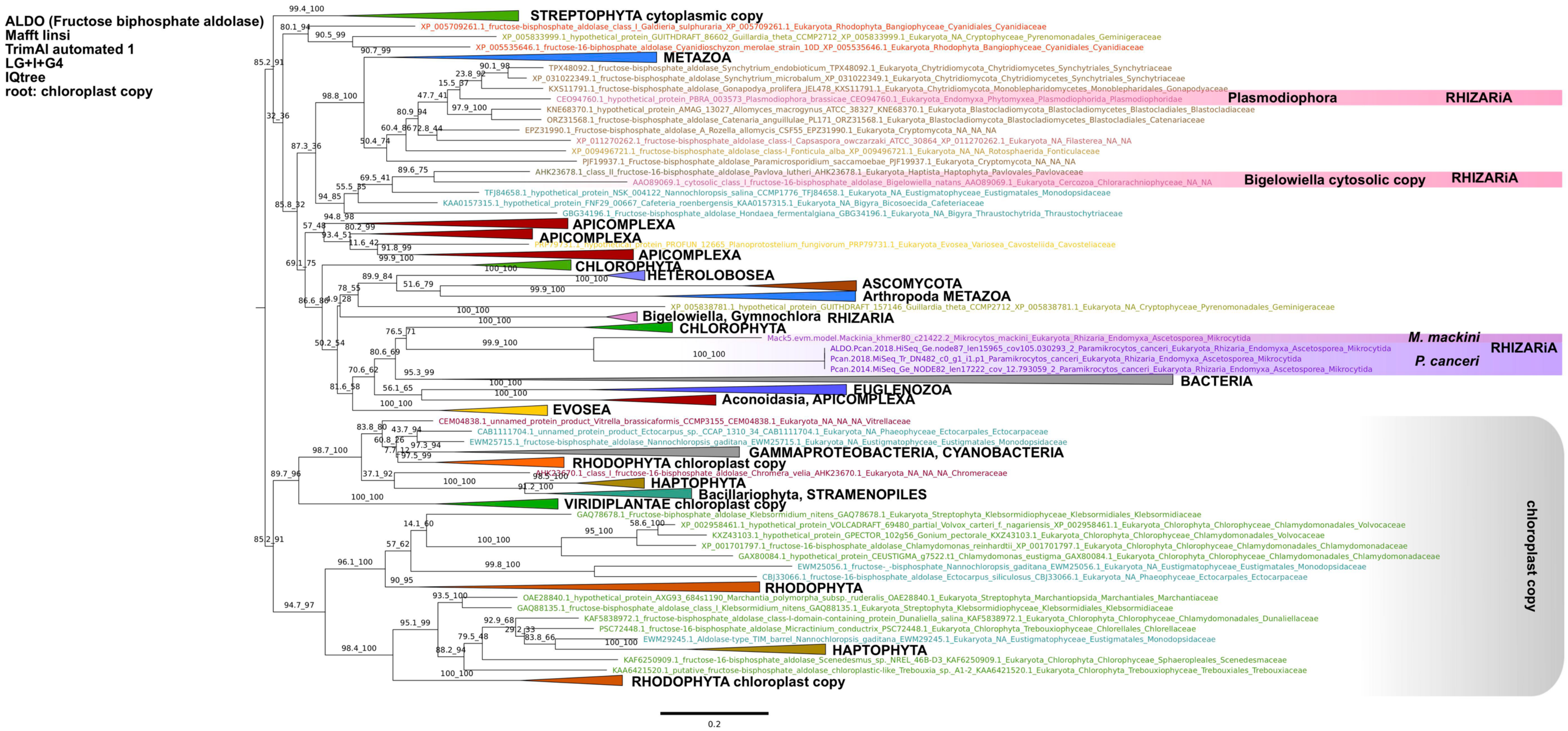

Fig. S5. Glycolysis pathway - preparatory phase – Step 5: TPI (triphosphate isomerase)

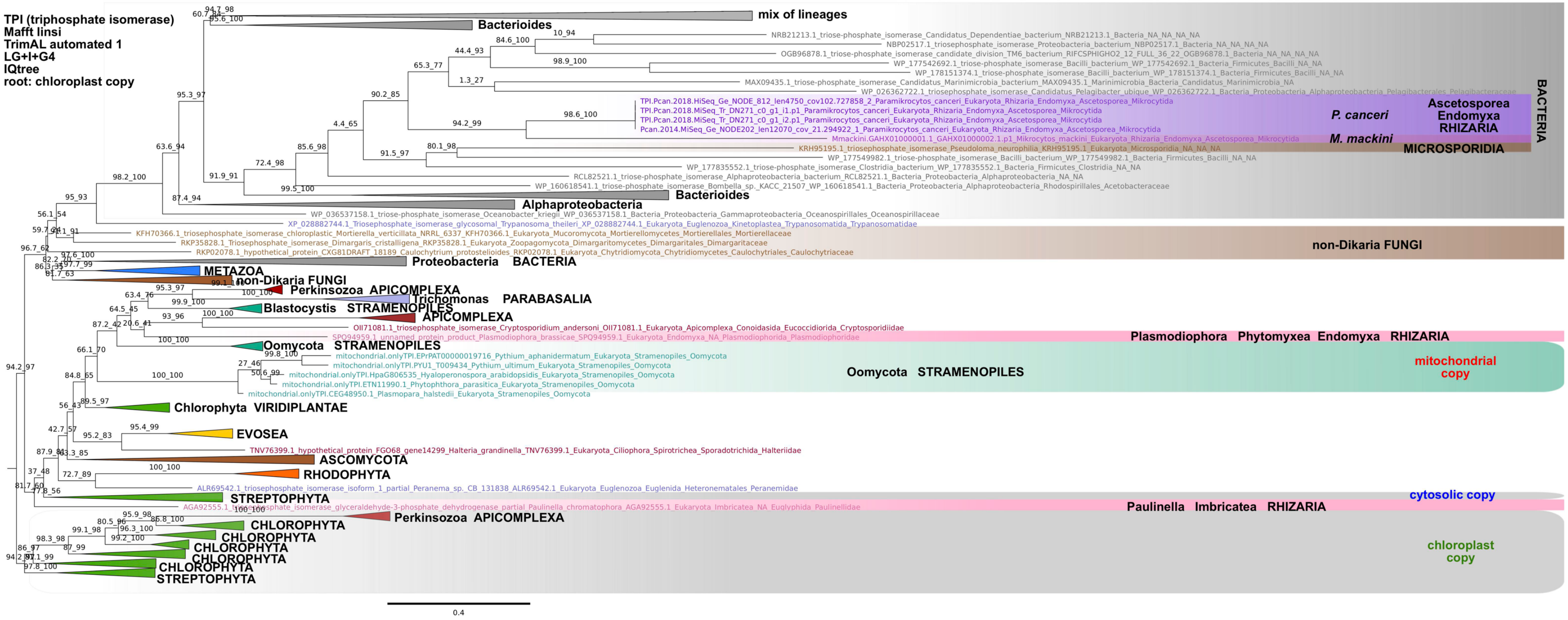

Fig. S5. Glycolysis pathway - pay off phase – Step 6: GAPDH (Glyceraldehyde 3-phosphate dehydrogenase)

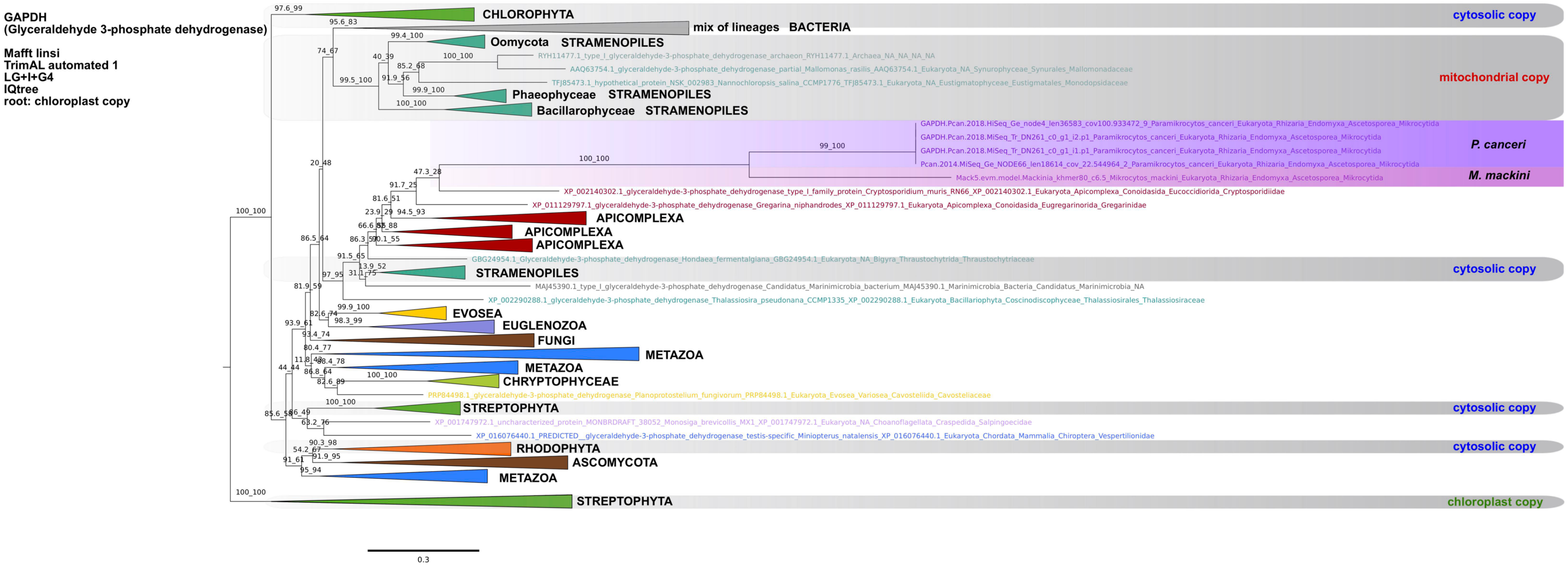

Fig. S5. Glycolysis pathway - pay off phase – Step 7: PGK (phosphoglycerate kinase)

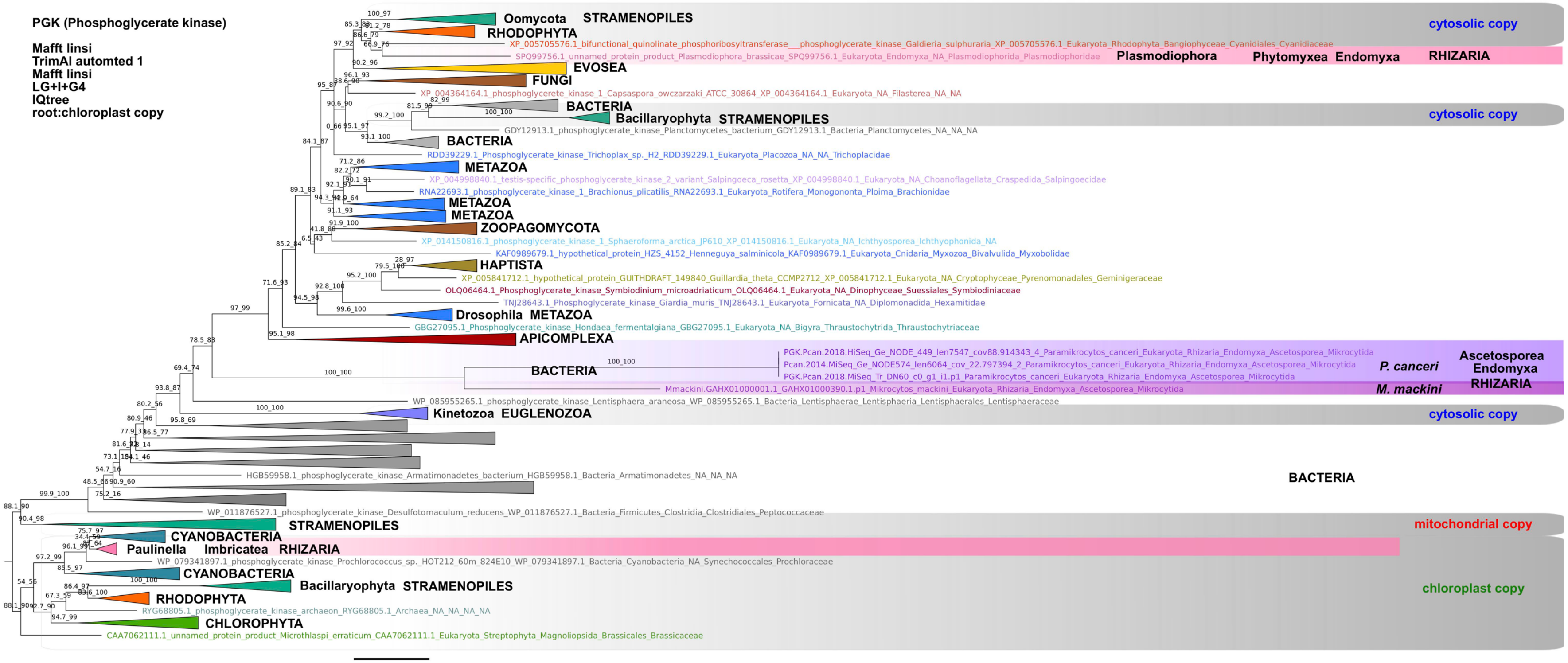

Fig. S5. Glycolysis pathway - pay off phase – Step 8: cPGM (cytosolic co-factor independent phosphoglycerate mutase)

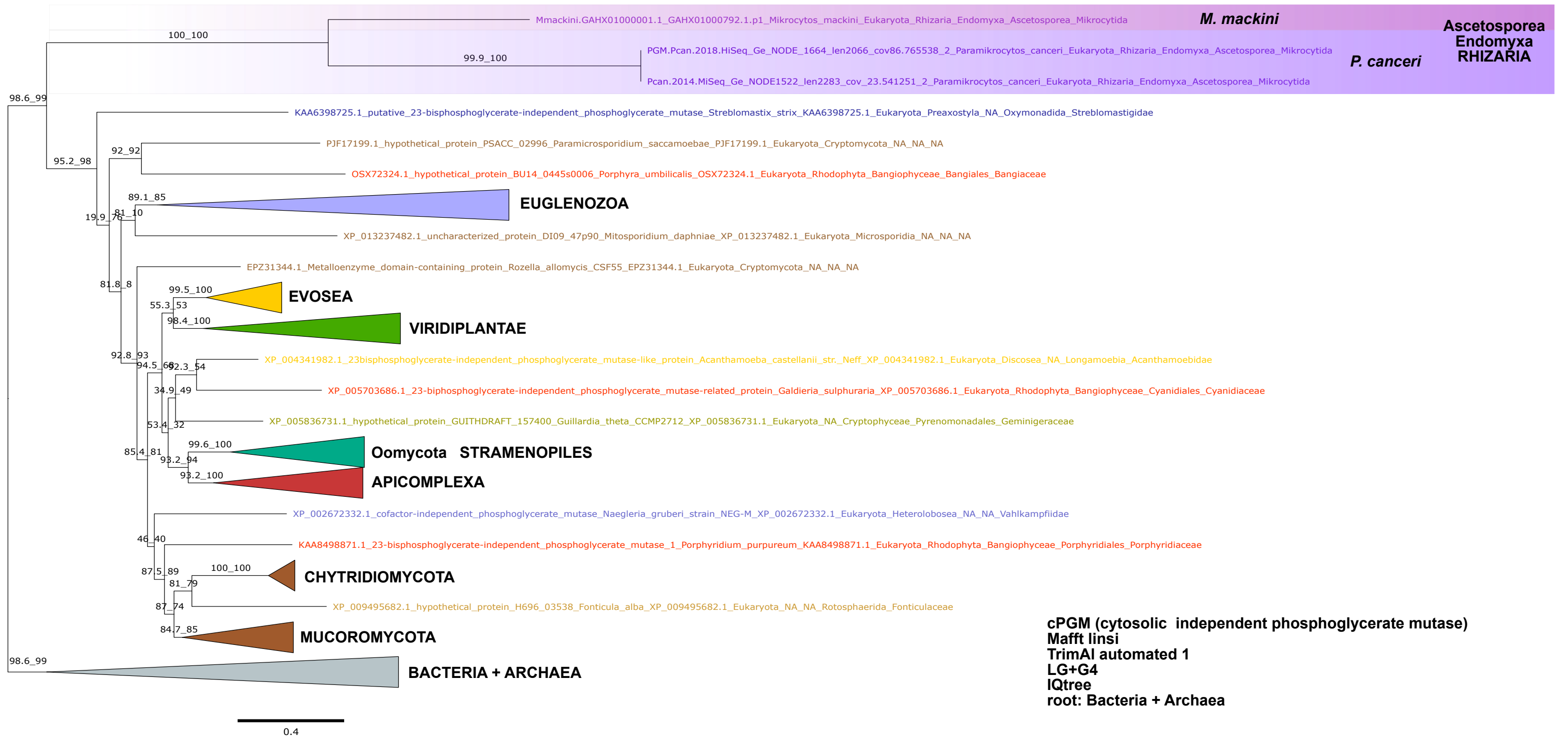

Fig. S5. Glycolysis pathway - pay off phase – Step 8: mPGM (mitochondrial co-factor independent phosphoglycerate mutase)

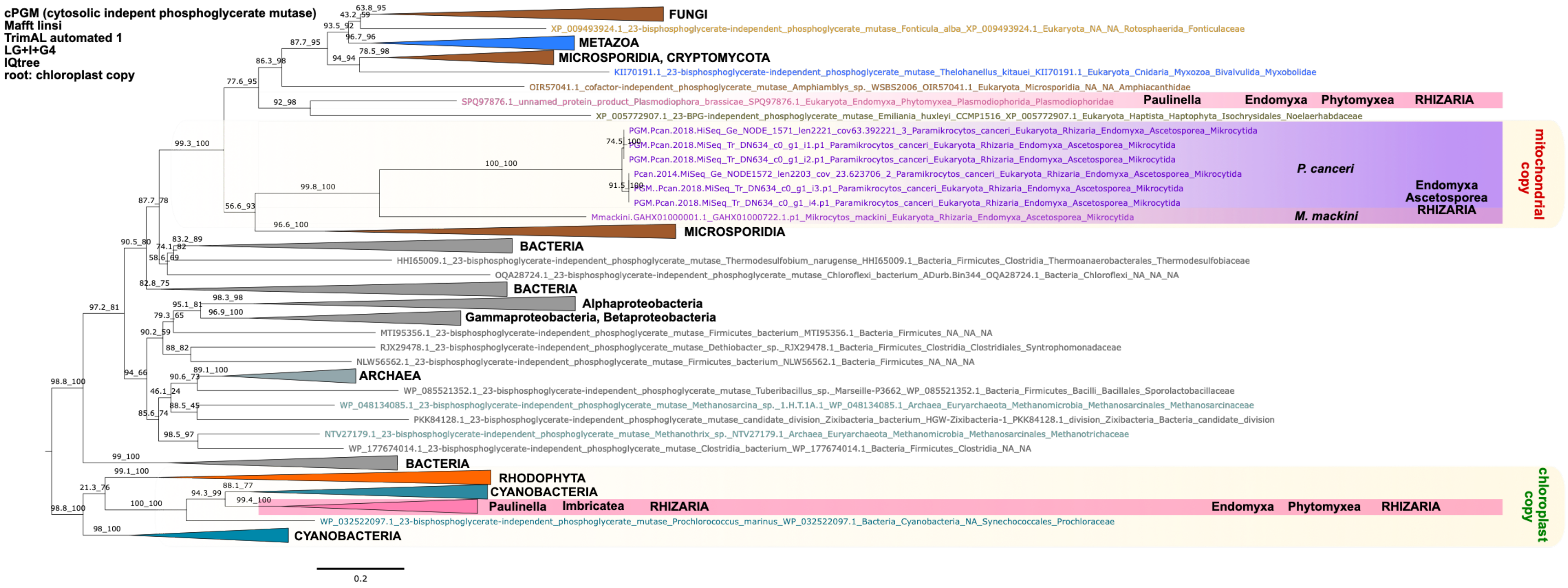

Fig. S5. Glycolysis pathway - pay off phase – Step 9: cENO (cytosolic enolase)

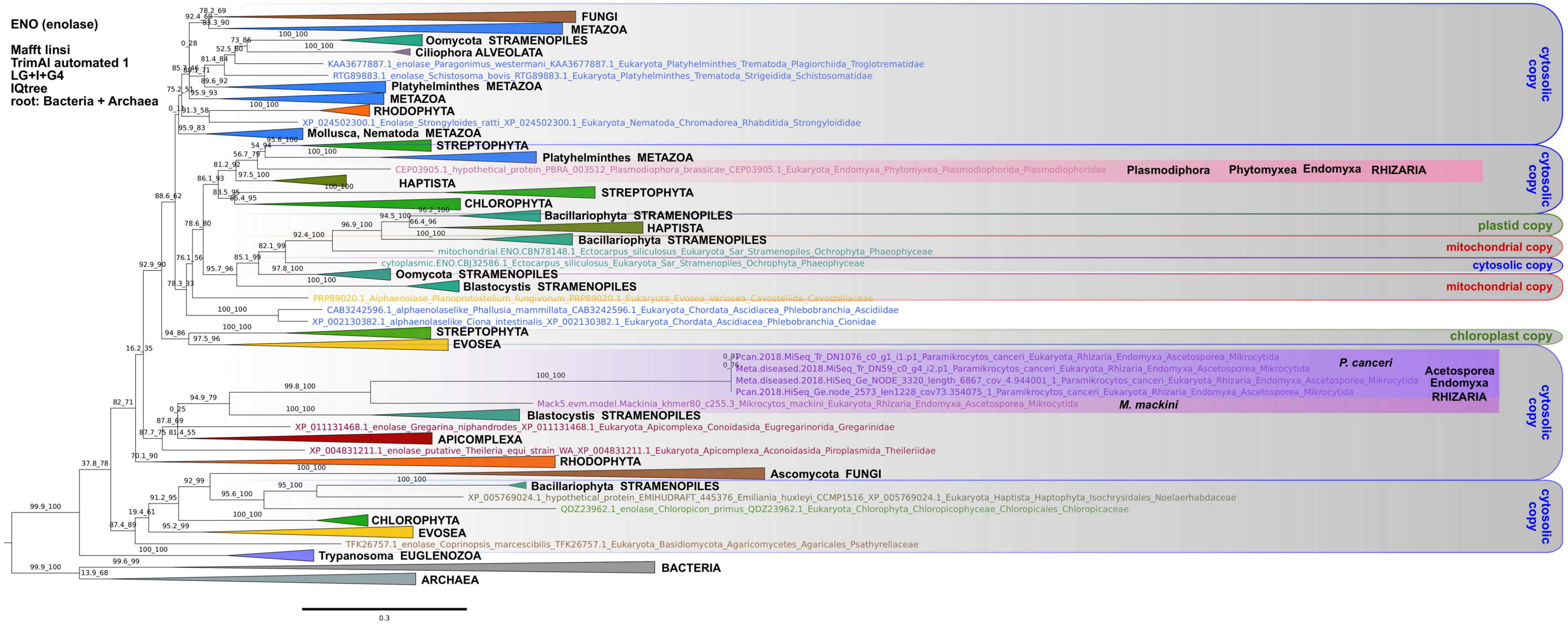

Fig. S5. Glycolysis pathway - pay off phase – Step 10: PK (pyruvate kinase)

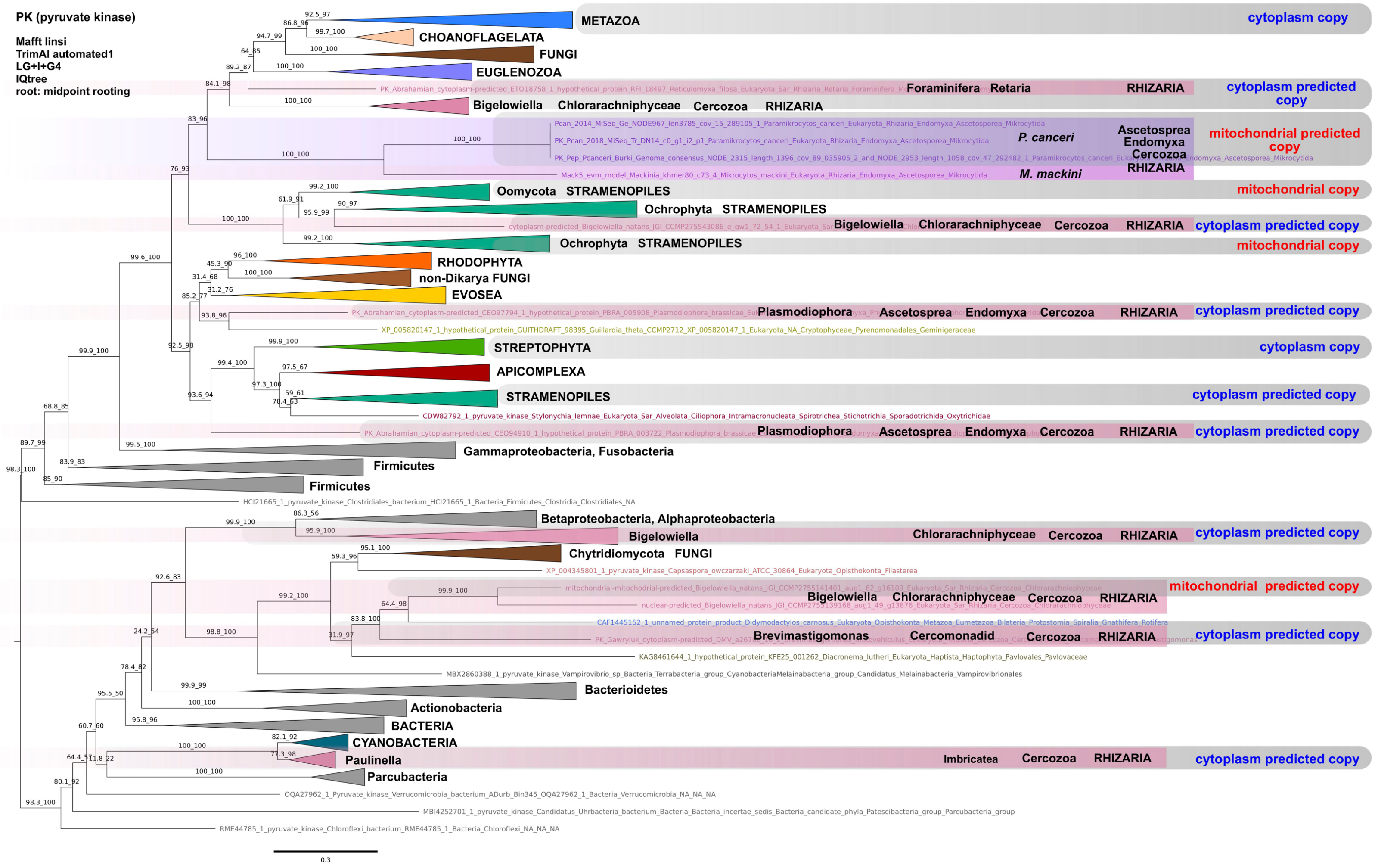

Fig. S5. CDP-DAG pathway: LPCAT (Lysophosphatidylcholine acyltransferase)

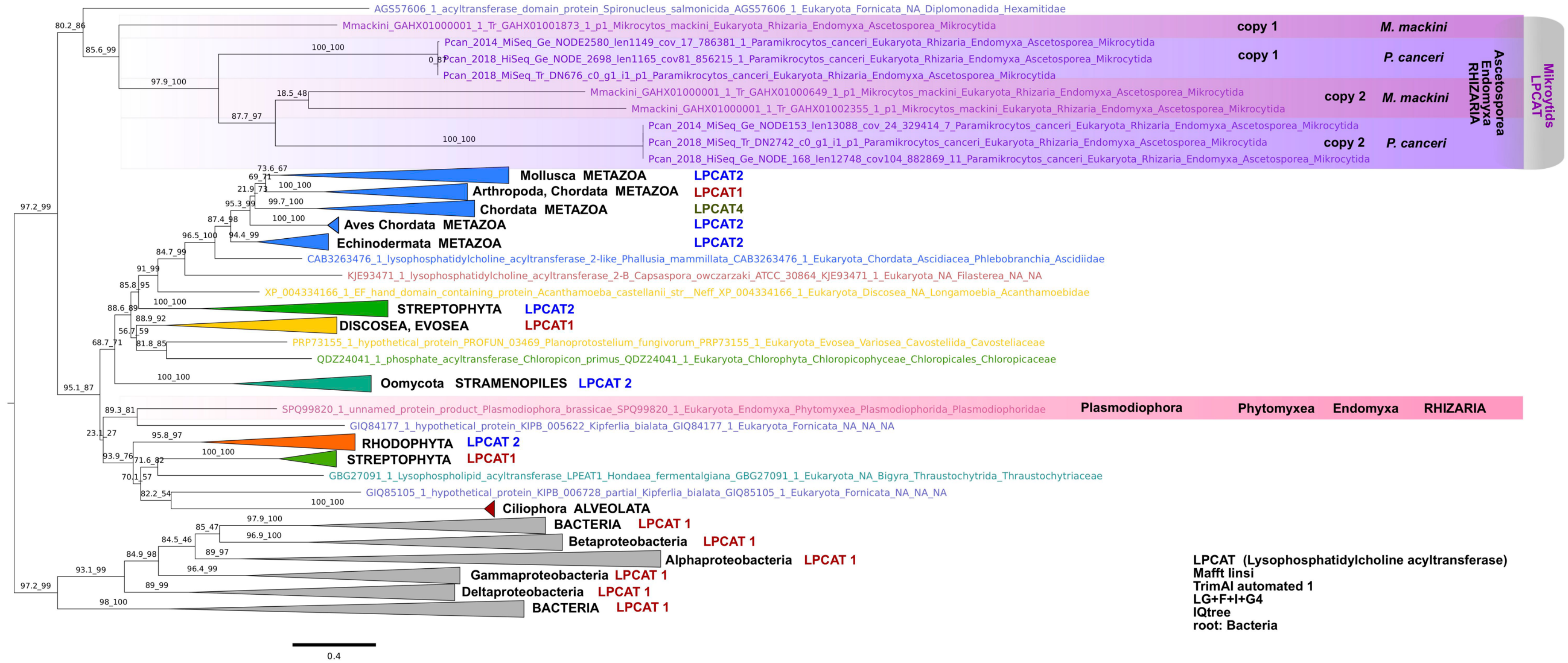

Fig. S5. CDP-DAG pathway: PSD (phosphatidylserine decarboxylase)

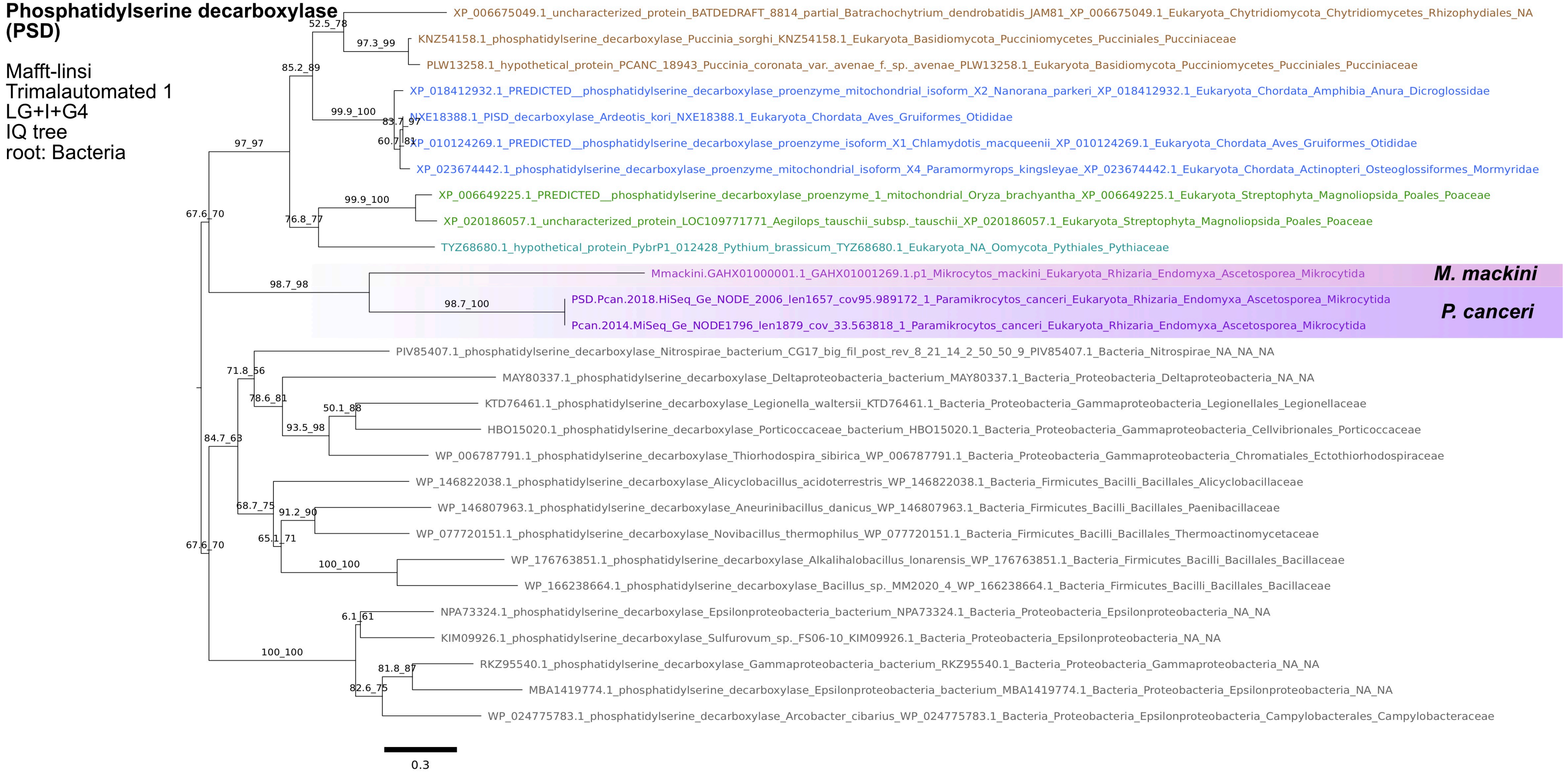

Fig. S5. CDP-DAG pathway: PSS2 (CDP-diacylglycerol--serine O-phosphatidyltransferase 2)

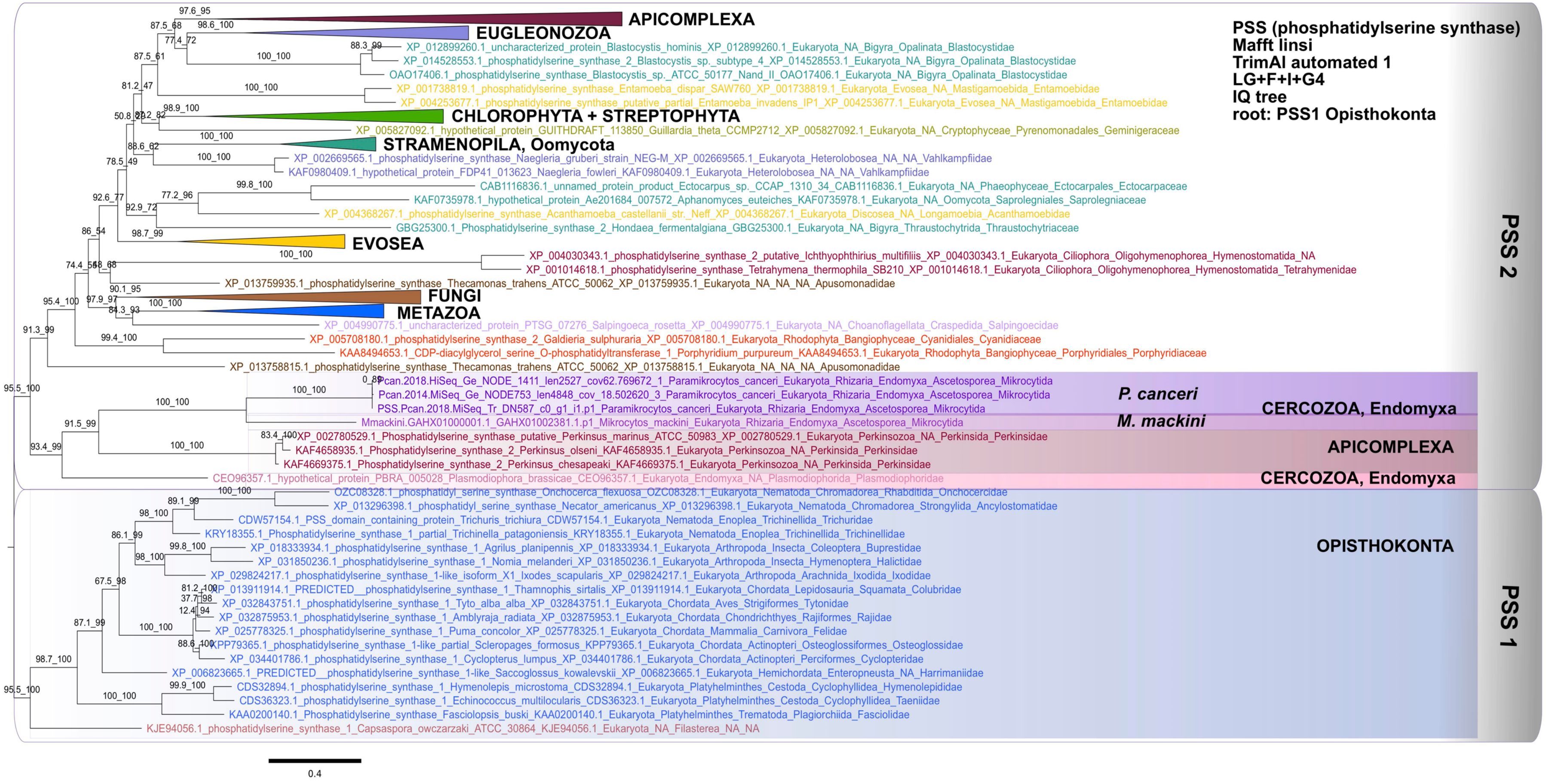

Fig. S5. ISC pathway for Fe-S cluster biosynthesis: Yah1 (Yeast Adrenodoxin Homolog)

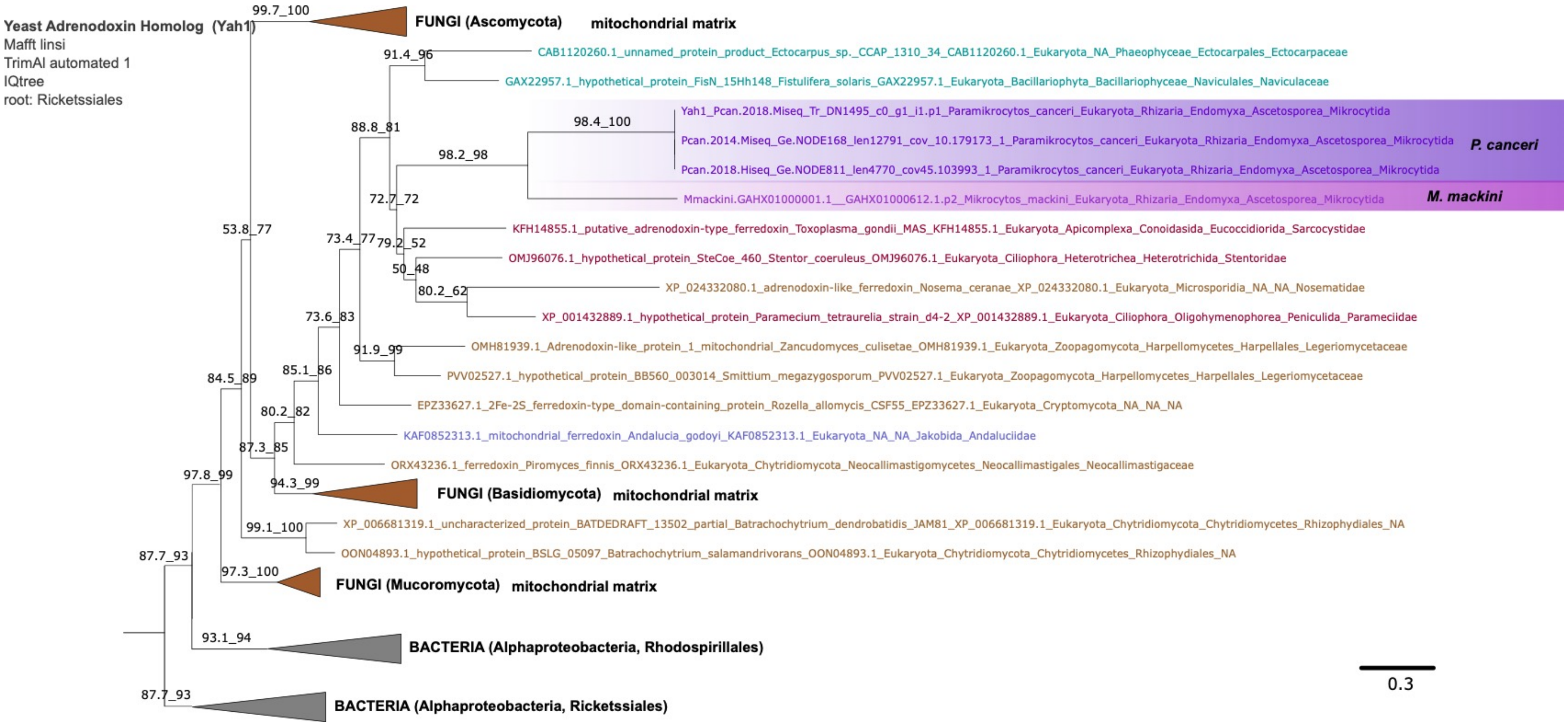

Fig. S5. ISC pathway for Fe-S cluster biosynthesis: FdxR (Ferredoxin Reductase)

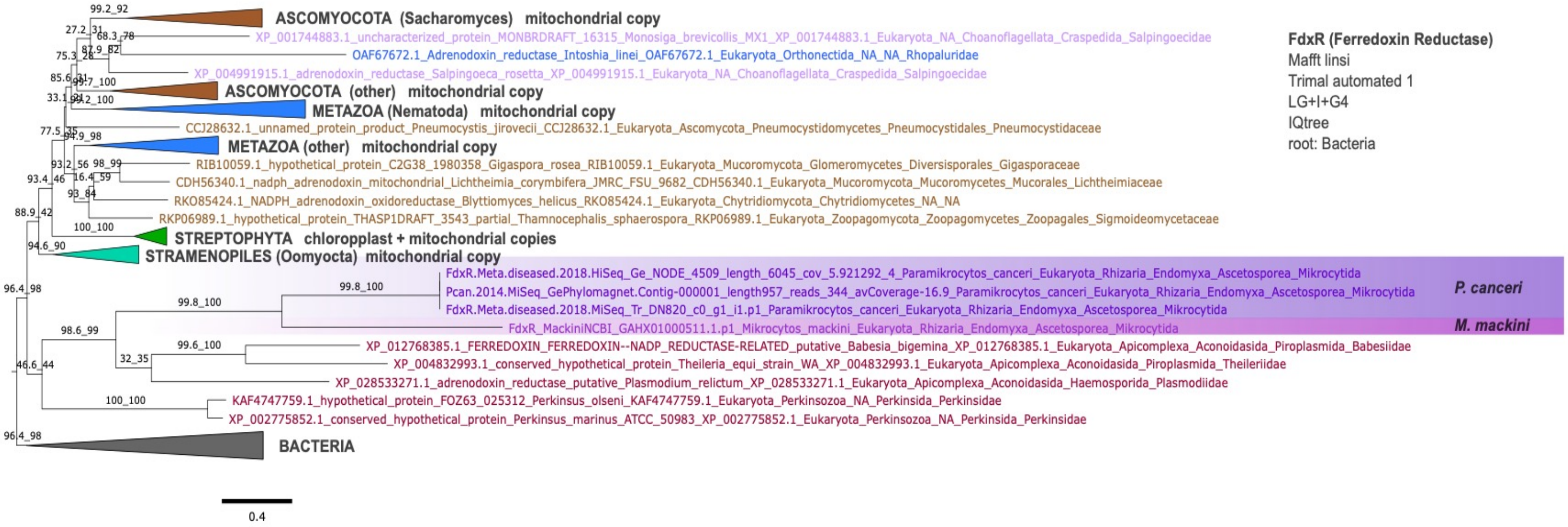

Fig. S5. ISC pathway for Fe-S cluster biosynthesis: IscS (Cysteine desulfurase)

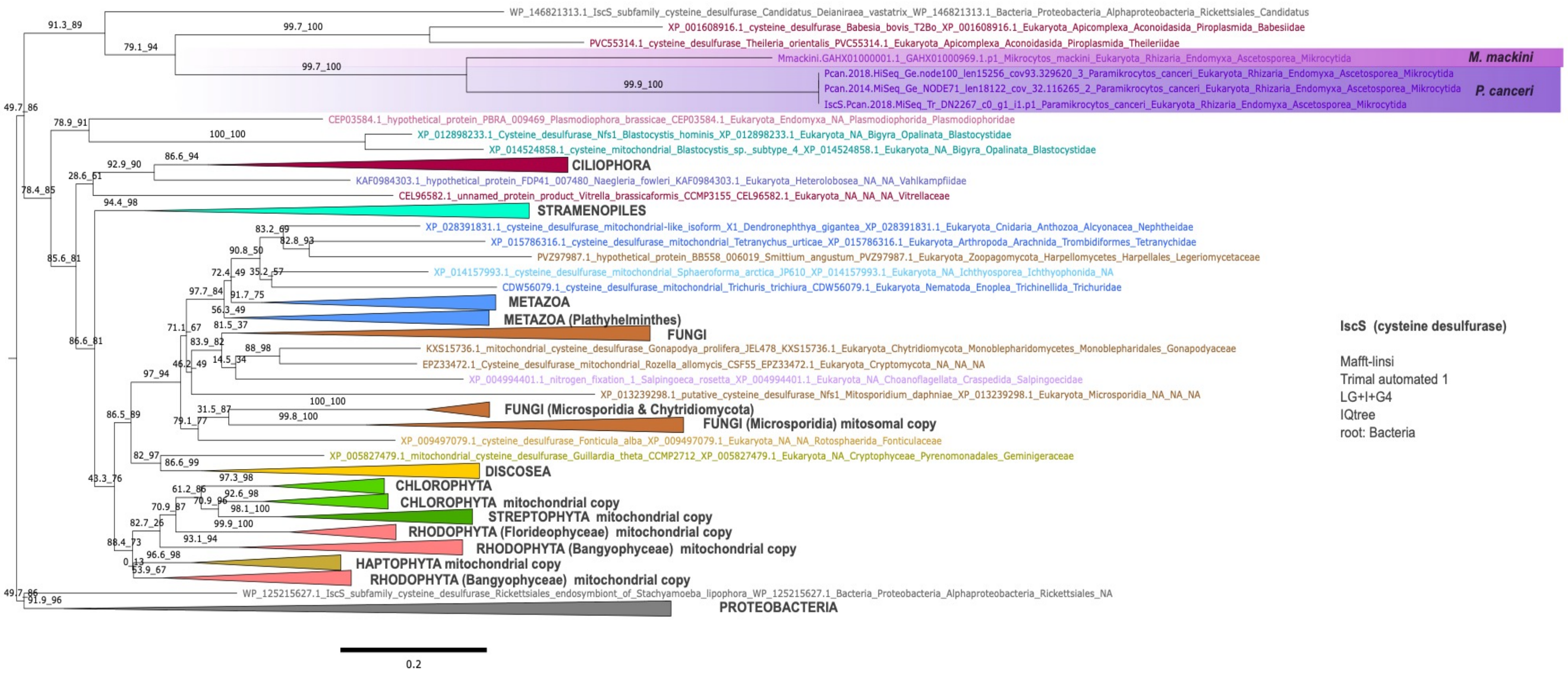

Fig. S5. ISC pathway for Fe-S cluster biosynthesis: CyaY (frataxin)

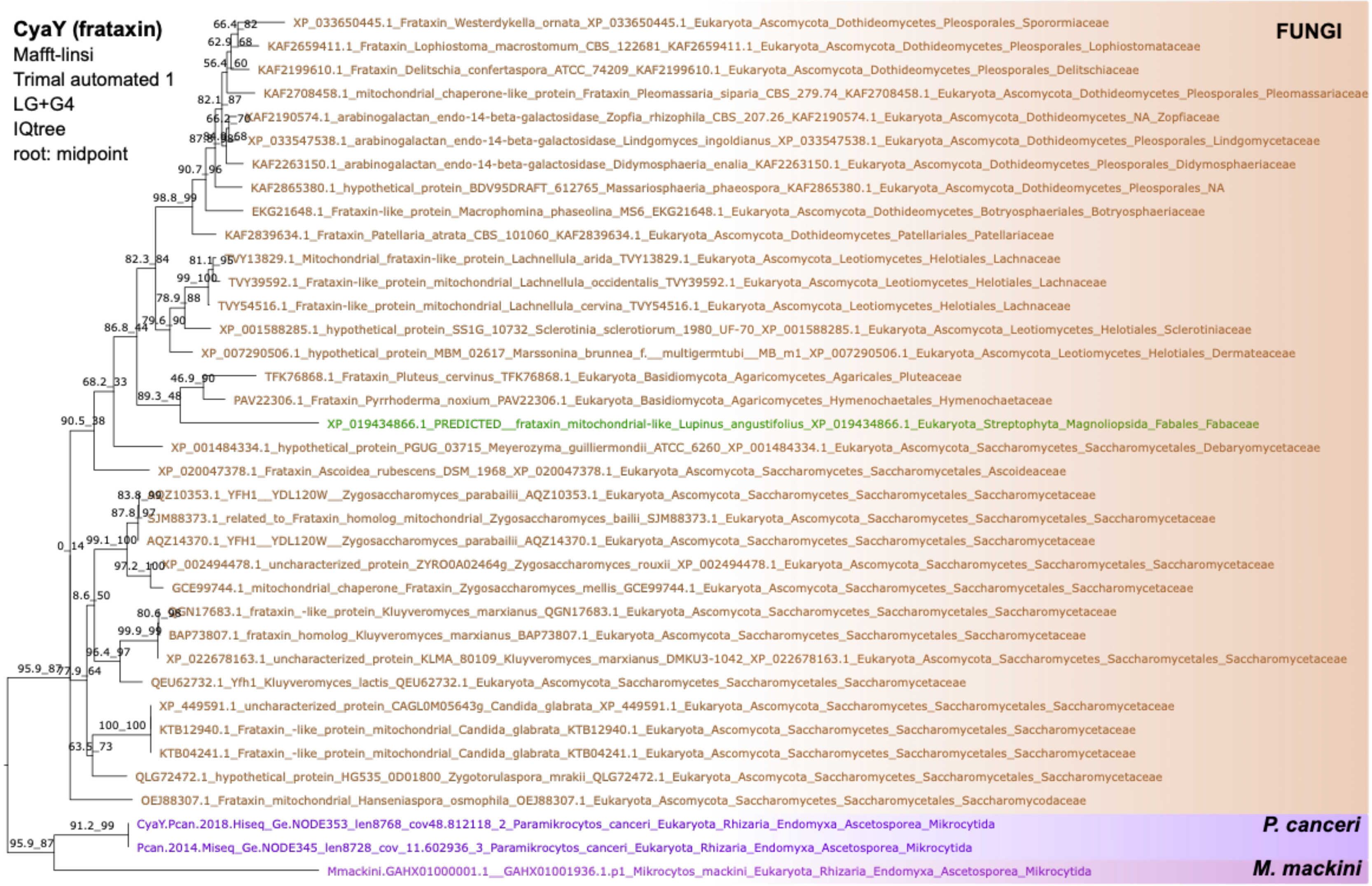

Fig. S5. ISC pathway for Fe-S cluster biosynthesis: HscB (Fe-S cluster chaperone)

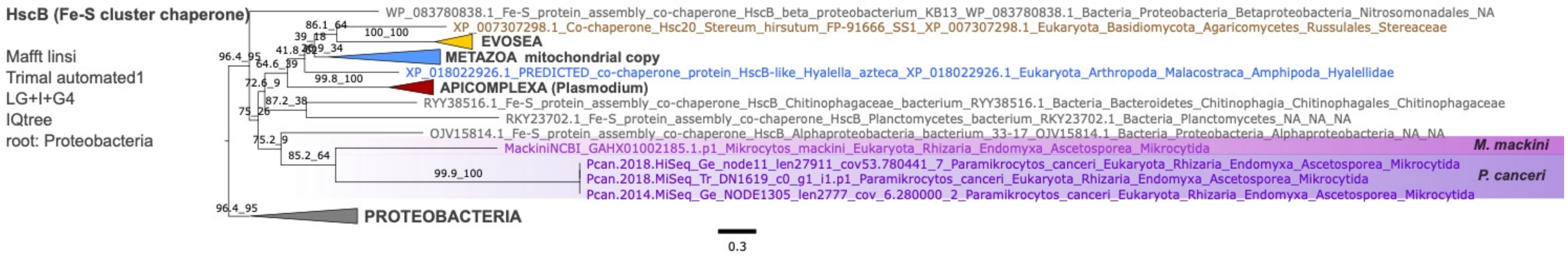

Fig. S5. ISC pathway for Fe-S cluster biosynthesis: IscU (Iron-Sulfur Cluster Assembly Enzyme)

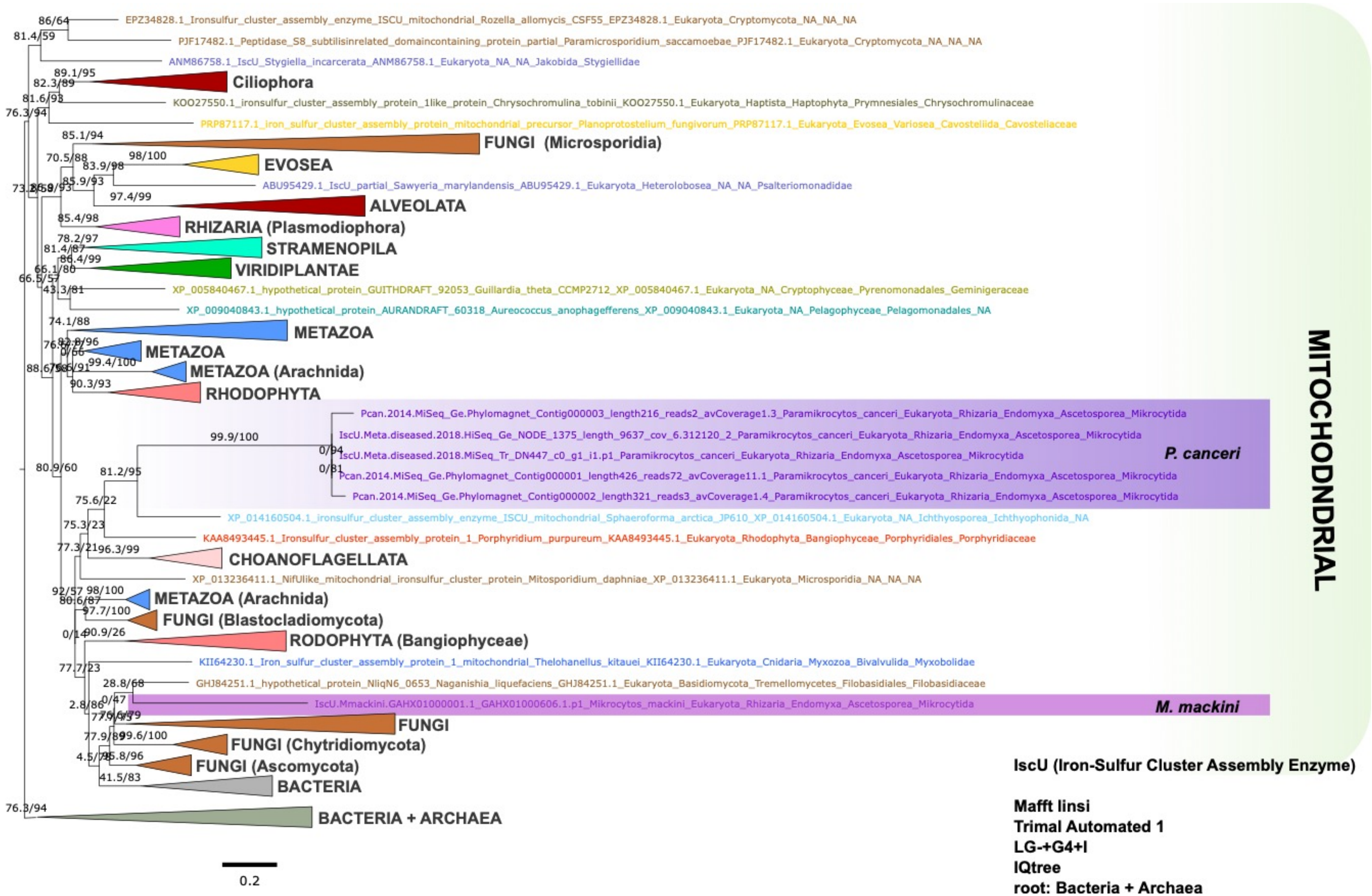

Fig. S5. ISC pathway for Fe-S cluster biosynthesis transporter: Atm1 (Iron-sulfur clusters transporter)

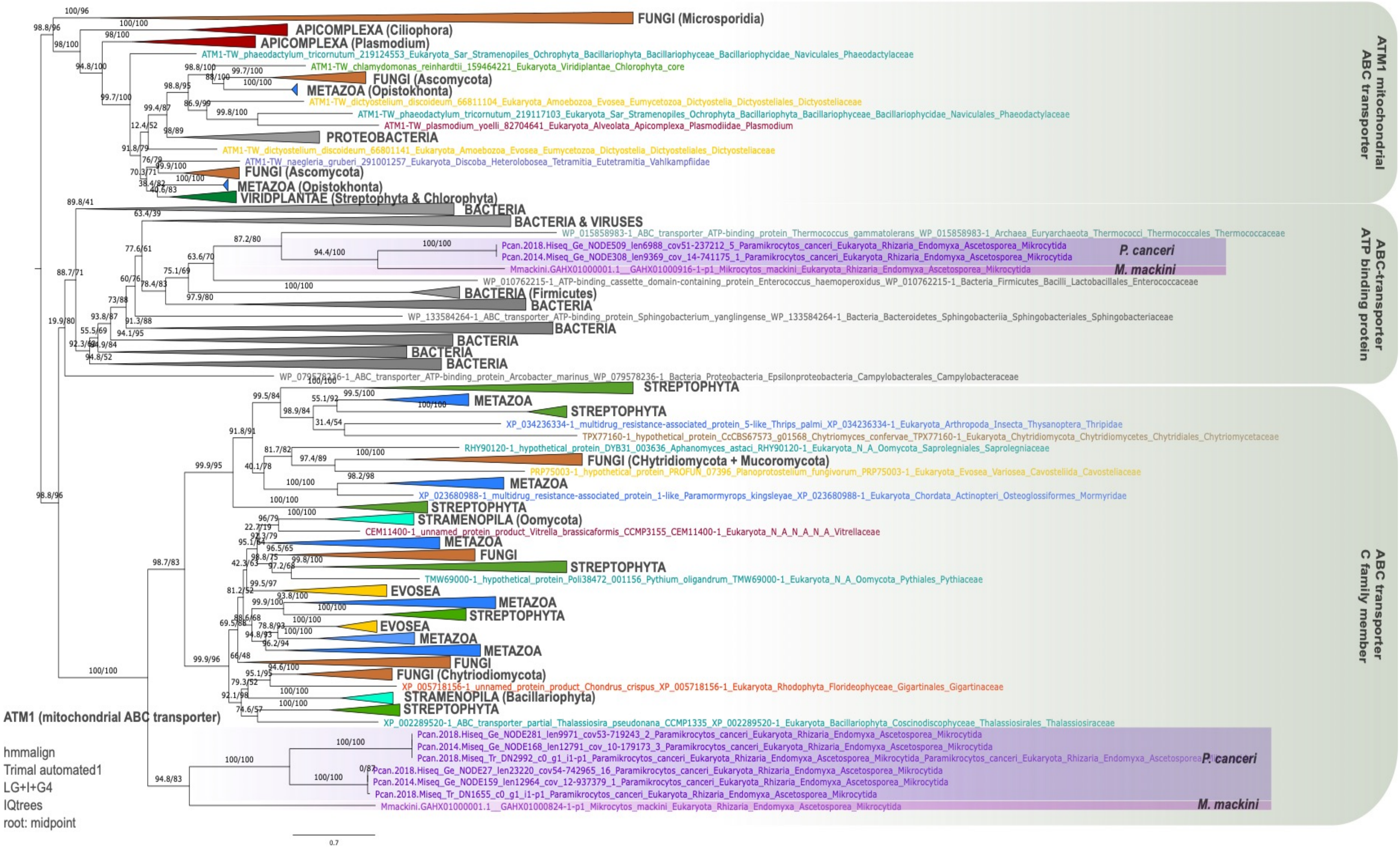

Fig. S5. Protein folding machinery: Hsp70 (70 kilodalton heat shock proteins)

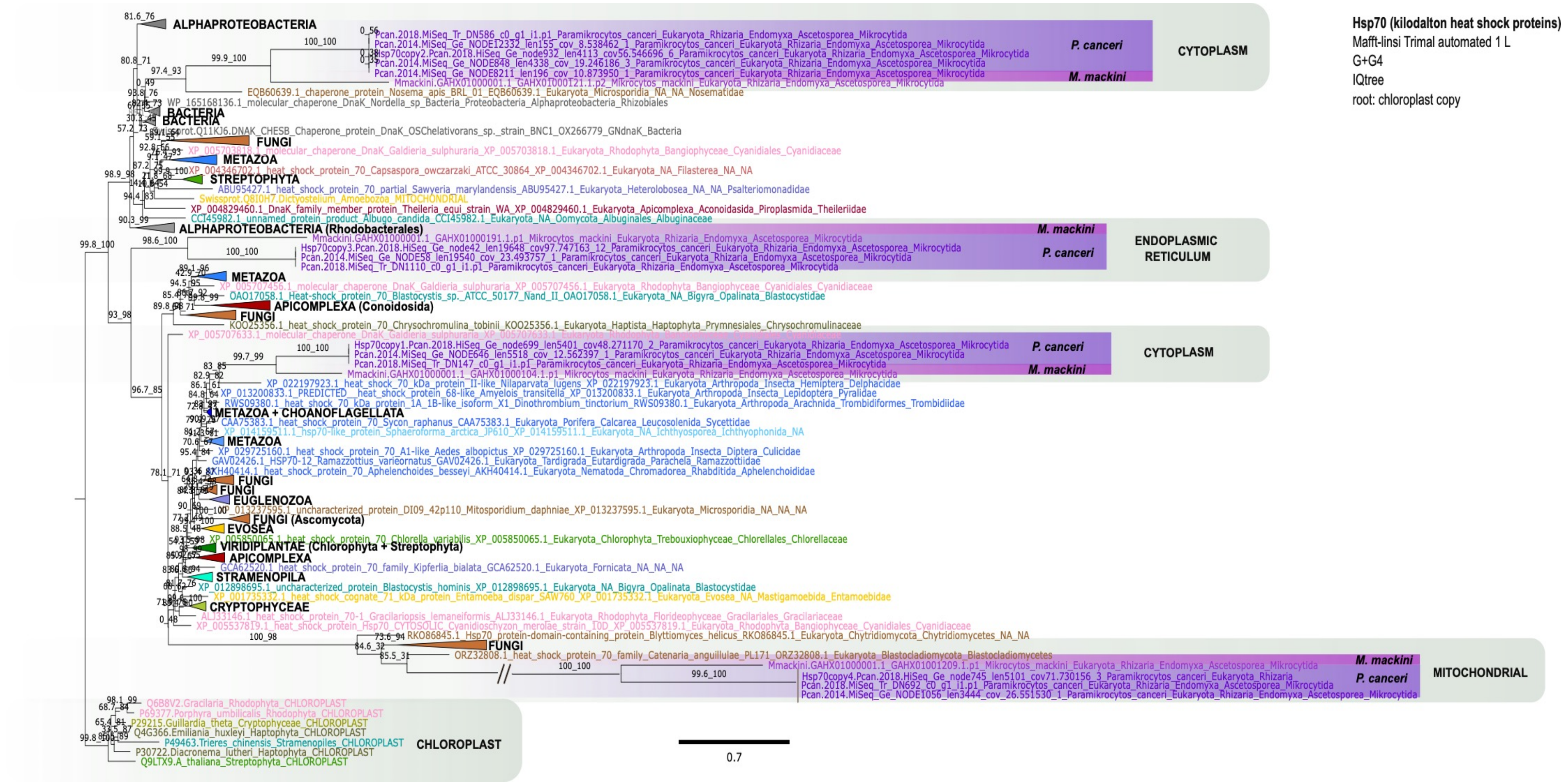

Fig. S5. Protein folding machinery: Hsp 90 (90 kilodalton heat shock proteins)

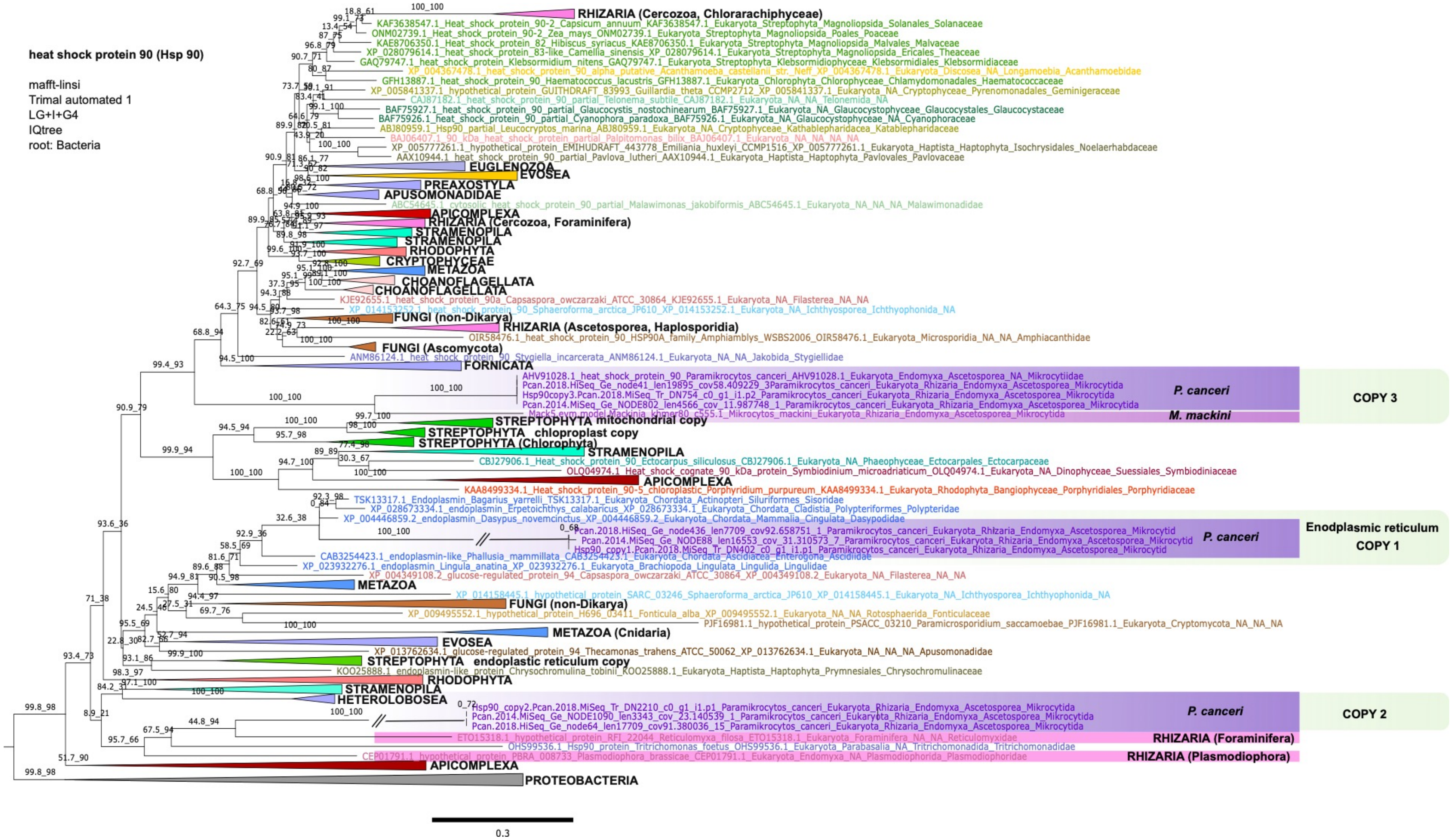

Fig. S5. Protein folding machinery: DnaJ (Chaperone J)

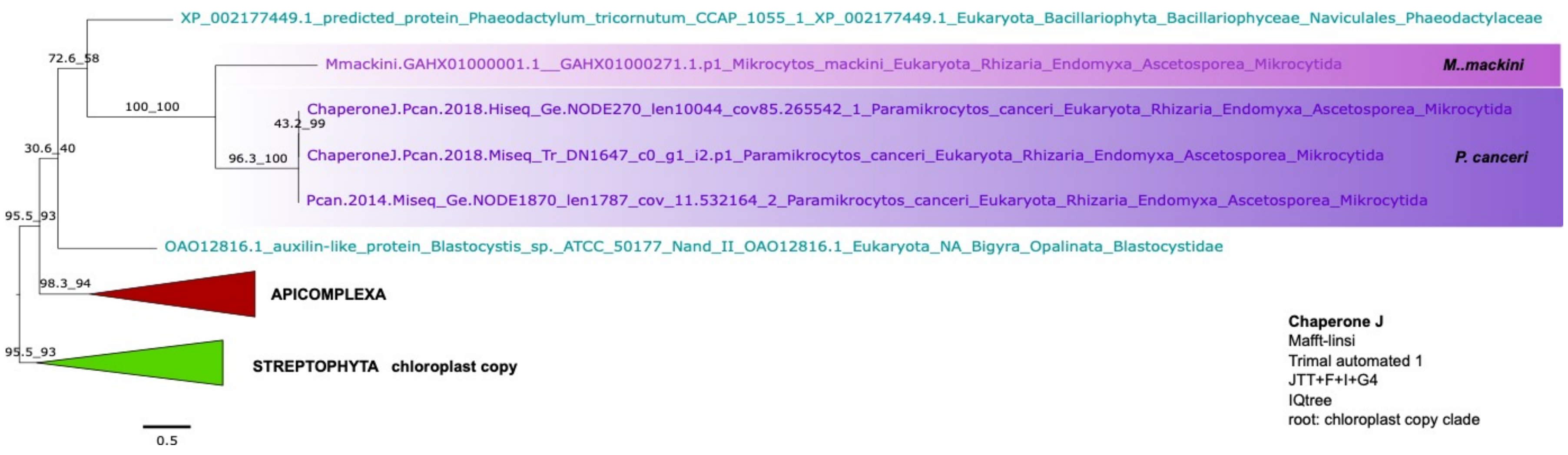

Fig. S5. Phylogeny of *P. canceri* mLDH (mitochondrial lactate dehydrogenase)

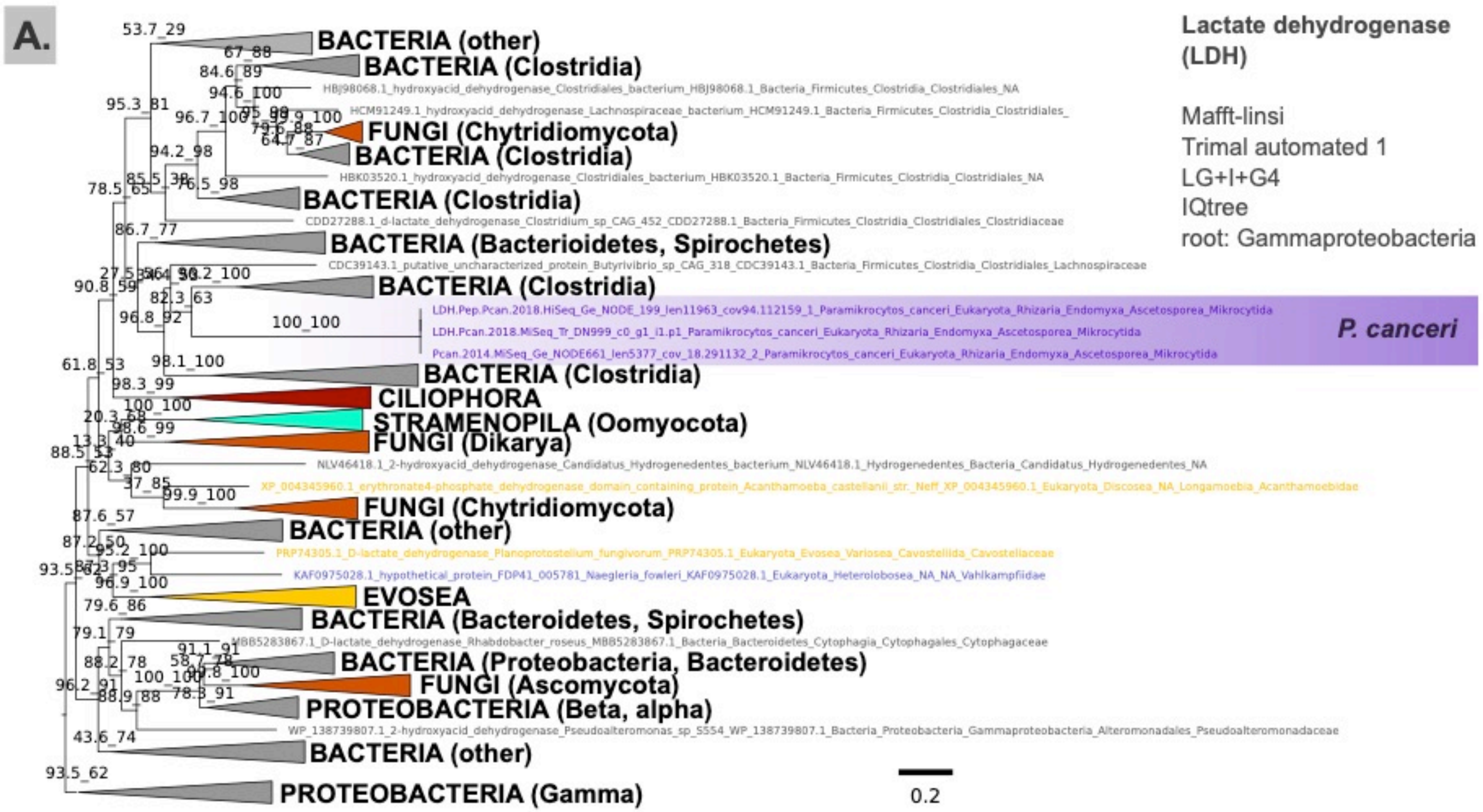

Figure S6 – Heterologous expressed green fluorescent protein (GFP) fusion proteins of *Paramikrocytos canceri* PSS and ISCU localize to yeast mitochondria. Yeast cells expressing (A) ISCU-GFP, (B) and (C) and PSS-GFP and empty plasmid pDDGFP were stained with mitochondrion-reactive stain Mitotracker CMXRos (red) and DNA stain DAPI (blue) and visualized by fluorescent microscopy. Not all yeast cells are transformed. Images represent a composite of multiple Z-slices, see methods for details. Scale bars represent 5 µm.

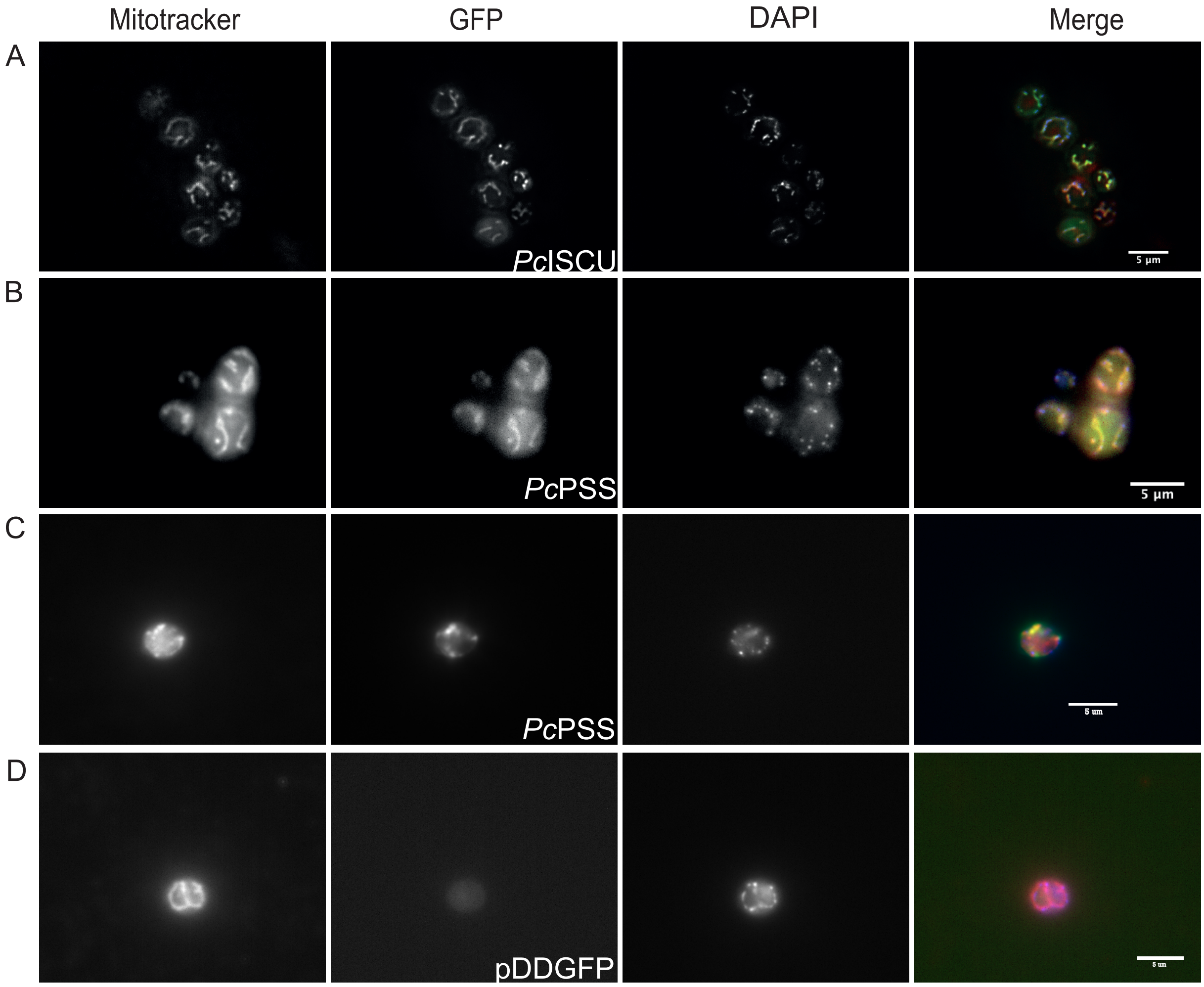

Fig. S7. Blob plots of the metagenomic assemblies of healthy and diseased tissues. Contigs are represented as circles. The size of the circles mirrors the contig size, while the color show contig taxonomic identity obtained through blast searches. For many contigs no homologues were identified on NCBI (gray circles). The GC profile is shown in the upper part of each graph and the read coverage profile to the right. (**A**) Blob profile of the metagenomic assembly of the crab healthy tissue **(B)** Blob profile of the metagenomic assembly of the crab diseased tissue. For (A) and (B) the blast searches were performed against NCBI nucleotide database and *P.canceri* genome assembly 1. The contigs of *P. canceri* are highlighted in purple. **(C)** Blob profile of the *P. canceri* assembly 1 obtained using the bioinformatic workflow.

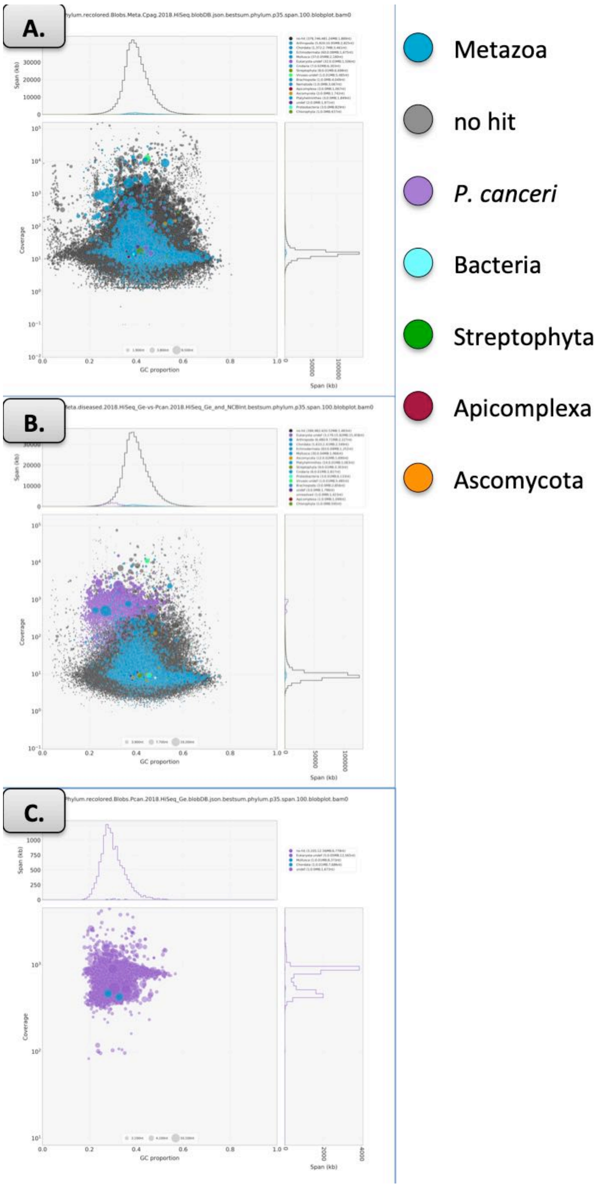

Table. S1. Related to Figure 1. Total genes identified in *P. canceri* and *M. mackini* assemblies

The table can be found in online in a separate excel file.

Table S2 Information about accession number and other quality control statistics for the DNA and RNA libraries used in this study

name of the Illumina library	Accession number	read library	host	type of tissue	PE reads**	read GC %
healthy tissue DNA.HiSeq_2018	ERR6474676	metagenomic DNA (1)	*Cancer pagurus*	healthy, claw muscle	2 x 84041113	40
healthy tissue cDNA.MiSeq_2018	ERR6496564	metranscriptomic cDNA (1)	*Cancer pagurus*	healthy, claw muscle	2 x 8524575	49
diseased tissue DNA.HiSeq_2018	ERR6496855	metagenomic DNA (1)	*Cancer pagurus*	diseased, antennal gland	2 x 112629383	34
diseased tissue cDNA.MiSeq_2018	ERR6496856	metranscriptomic cDNA (1)	*Cancer pagurus*	diseased, antennal gland	2 x 8158728	48
diseased tissue DNA.HiSeq_2014	ERR6496965	metagenomic DNA (2)	*Cancer pagurus*	diseased, antennal gland	2 x 7093938	36
P. canceri DNA.HiSeq_2018	ERR6496897	decontaminated DNA in START 1*	removed	-	2 x 47955599	30
P. canceri cDNA.MiSeq_2018	ERR6496963	decontaminated cDNA in START 3*	removed	-	2 x 5312634	48
P. canceri DNA.MiSeq_2014	ERR6497049	decontaminated DNA in START 2*	removed	-	2 x 2375278	32

* see Figure 1 of the bioinformatic workflow; ** number of raw reads after trimming and adapter removal (FastQC output);

(1) read library sequenced for this study; (2) library sequenced by Hartikainen *et al* 2014;
